# DNA Methylation and Expression of the *EgDEF1* Gene and Neighboring Retrotransposons in *mantled* Somaclonal Variants of Oil Palm

**DOI:** 10.1371/journal.pone.0091896

**Published:** 2014-03-17

**Authors:** Estelle Jaligot, Wei Yeng Hooi, Emilie Debladis, Frédérique Richaud, Thierry Beulé, Myriam Collin, Mawussé D. T. Agbessi, François Sabot, Olivier Garsmeur, Angélique D'Hont, Sharifah Shahrul Rabiah Syed Alwee, Alain Rival

**Affiliations:** 1 UMR DIADE, CIRAD, Montpellier, France; 2 UMR DIADE, IRD, Montpellier, France; 3 UMR AGAP, CIRAD, Montpellier, France; 4 FELDA Biotechnology Centre, FASSB, Bandar Enstek, Malaysia; Ecole Normale Superieure, France

## Abstract

The *mantled* floral phenotype of oil palm (*Elaeis guineensis*) affects somatic embryogenesis-derived individuals and is morphologically similar to mutants defective in the B-class MADS-box genes. This somaclonal variation has been previously demonstrated to be associated to a significant deficit in genome-wide DNA methylation. In order to elucidate the possible role of DNA methylation in the transcriptional regulation of *EgDEF1*, the *APETALA3* ortholog of oil palm, we studied this epigenetic mark within the gene in parallel with transcript accumulation in both normal and *mantled* developing inflorescences. We also examined the methylation and expression of two neighboring retrotransposons that might interfere with *EgDEF1* regulation. We show that the *EgDEF1* gene is essentially unmethylated and that its methylation pattern does not change with the floral phenotype whereas expression is dramatically different, ruling out a direct implication of DNA methylation in the regulation of this gene. Also, we find that both the *gypsy* element inserted within an intron of the *EgDEF1* gene and the *copia* element located upstream from the promoter are heavily methylated and show little or no expression. Interestingly, we identify a shorter, alternative transcript produced by *EgDEF1* and characterize its accumulation with respect to its full-length counterpart. We demonstrate that, depending on the floral phenotype, the respective proportions of these two transcripts change differently during inflorescence development. We discuss the possible phenotypical consequences of this alternative splicing and the new questions it raises in the search for the molecular mechanisms underlying the *mantled* phenotype in the oil palm.

## Introduction

The first clue to the epigenetic origin of the *mantled* somaclonal variation in the oil palm (*Elaeis guineensis* Jacq.), which is only visible in the flowers and fruits of adult somatic embryo-derived palms, emerged from the observation of its highly variable incidence and severity combined with its ability to revert spontaneously [1]. Since then, the occurrence of alterations in DNA methylation patterns and gene expression in both *in vitro*-cultivated and adult variant tissues has been documented [2]–[7]. The strong resemblance between the morphology of the variant flowers (displaying a homeotic conversion of male floral organs into female ones) and mutants for B-class MADS-box genes involved in petal and stamen identity [8] led to the assumption that the expression of this gene subfamily could be affected in *mantled* palms. Indeed, previous studies from Adam *et al.* [9], [10] showed that the putative B-class gene orthologs identified in oil palm are globally downregulated in developing *mantled* inflorescences with respect to their normal counterparts. Among these genes, *EgDEF1*, which is most similar to *Arabidopsis thaliana*'s *APETALA3* and *Antirrhinum majus*' *DEFICIENS*, undergoes the strongest decrease in transcript accumulation in both male and female *mantled* inflorescences at all developmental stages [10]. What remains to be determined is whether DNA methylation, which is significantly impaired in *mantled* palms inflorescences, could also be involved in the downregulation of this organ identity gene in the context of the variant phenotype. Such a correlation has been found in *Arabidopsis thaliana* mutants that are depleted in genome-wide DNA methylation while displaying both targeted methylation-based transcriptional silencing of MADS-box genes involved in floral morphogenesis and floral development defects [11], [12].

In the present work, we investigated DNA methylation within the proximal promoter sequence and the start of the coding sequence of the *EgDEF1* gene through both bisulfite sequencing and McrBC-PCR approaches. The identification of the complete genomic sequence of the *EgDEF1* gene revealed the presence of an oversized fifth intron and the occurrence of two Transposable Elements (TEs) located respectively within the enlarged intron of *EgDEF1* and upstream of its putative promoter sequence. Although most TEs are maintained in a “default” silenced state by the host genome, they can be reactivated in methylation-defective backgrounds or in stress conditions, such as *in vitro* culture, and alter the expression of neighboring genes [13]–[15]. Alternatively, the repressive epigenetic marks targeting the TEs can “spread” toward adjacent genes, impairing their expression [16], [17]. In order to explore the possibility of an interference of TE regulation on gene expression in the context of the *mantled* floral phenotype, we investigated the DNA methylation of the two elements and their expression was estimated by real-time quantitative PCR (rt-qPCR). Finally, we hypothesized that the large size of *EgDEF1*'s intron 5 could, in some cases, induce aberrant transcription or splicing events, ultimately leading to the production of shorter transcripts. We assessed this possibility by using 3′-RACE and the accumulation pattern of one such alternative transcript was examined in developing inflorescences by rt-qPCR. In the aim of better understanding the potential of the truncated transcript to interfere with its full-length counterpart, we compared their respective abundances within each inflorescence stage between the two floral phenotypes by performing absolute quantitation of each transcript. While the molecular mechanisms underlying this alternative transcription are still unknown, our findings point to interesting new directions for research on the *mantled* variation.

## Material and Methods

### Ethics statement

The sampling of the plant material used in the experiments was covered by Material Transfer Agreements (MTAs) between the involved Institutions.

### Plant material and histological analyses

Both male and female immature inflorescences originating from normal and *mantled* clonally propagated *tenera* (*dura* x *pisifera*) oil palms [18] were sampled at FELDA (Federal Land Development Authority, Malaysia) Tun Razak Estate and at the LaMé CNRA (Centre National de la Recherche Agronomique) Principal Research Station in Côte d'Ivoire. Clonal palms originating from the same mother palm, regenerated under the same somatic embryogenesis-based protocol and planted on the same date in the same plot were selected. The genetic origin of plant material used in the present study is given in Table S1.

Inflorescence series were obtained by sampling all the inflorescences of a given palm located between leaves of order +4 and +18 (the youngest expanding leaf being of order 0) and flowers were dissected as described previously [19]. One half of the dissected flower tissues was either flash-frozen in liquid nitrogen or immersed in RNAlater solution (Ambion), then stored at either −80°C or −20°C until processed for the extraction of nucleic acids. In parallel, the remaining half was fixated for future histological analyses through two times 5 minutes vacuum infiltration in 2× PBS, 4% paraformaldehyde buffer, then rinsed three times in 1× PBS. Dehydration was then achieved through two successive 1-h immersions in 50% then 70% ethanol. Histological analyses were performed as previously described [19] in order to determine the developmental stage of flower tissues. Stage 3 inflorescences from FC2317 palms were used in our methylation studies. Approximately 300 inflorescences sampled from 10 genotypically distinct normal/*mantled* regenerant palms were analyzed in order to identify near-complete developmental series (*i.e.* inflorescences of the same sex ranging from stage 0 to stage 5 and displayed on the same palm) for both sexes and within the same clonal line (FC2405). Samples used in our rt-qPCR experiments therefore reflect the range of inflorescence stages present on each palm at the time of sampling.

### Nucleic acids extraction and purification

Genomic DNA and/or total RNAs were extracted using the DNeasy Plant Mini Kit and the RNeasy Plant Mini Kit (Qiagen) respectively, according to the recommendations of the manufacturer. Purity and concentration of the eluted nucleic acids were estimated using a NanoDrop ND-100 spectrophotometer (Thermo Scientific) and their integrity was assessed by electrophoretic separation and visualization under UV light. Due to the instant oxidization occurring upon dissecting inflorescence tissues at stages 4 and 5, most of the corresponding RNA extracts were too degraded to be used in the subsequent qPCR experiments.

### Identification of the *EgDEF1* genomic sequence

Using the full-length cDNA sequence of *EgDEF1* (accession number AY739700.1) as a template for 5′-oriented primer design, we performed chromosome walking on genomic DNA sampled from a palm of FC166 origin using the GenomeWalker Kit with the Advantage 2 Polymerase (both from Clontech) according to the supplier's recommendations. This first step allowed us to recover a genomic fragment extending from nucleotide positions -1833 to +12 relatively to the ATG of the gene. This genomic fragment was then used as a probe to screen high density filters from an oil palm BAC genomic library constructed from a seed-derived individual of L2T origin [20]. Four positive clones showing identical hybridization patterns were isolated and one, named Eg133H20, was sequenced by Genoscope (Evry, France). Finally, the structure of the *EgDEF1* gene was confirmed through further chromosome walking on FC166 genomic DNA, using the primers shown in Table S2. Sequence similarity searches were performed using the BLASTN (version 2.2.27+ [21]) and BLASTX [22] programmes.

### Southern blot

**Figure 1:**
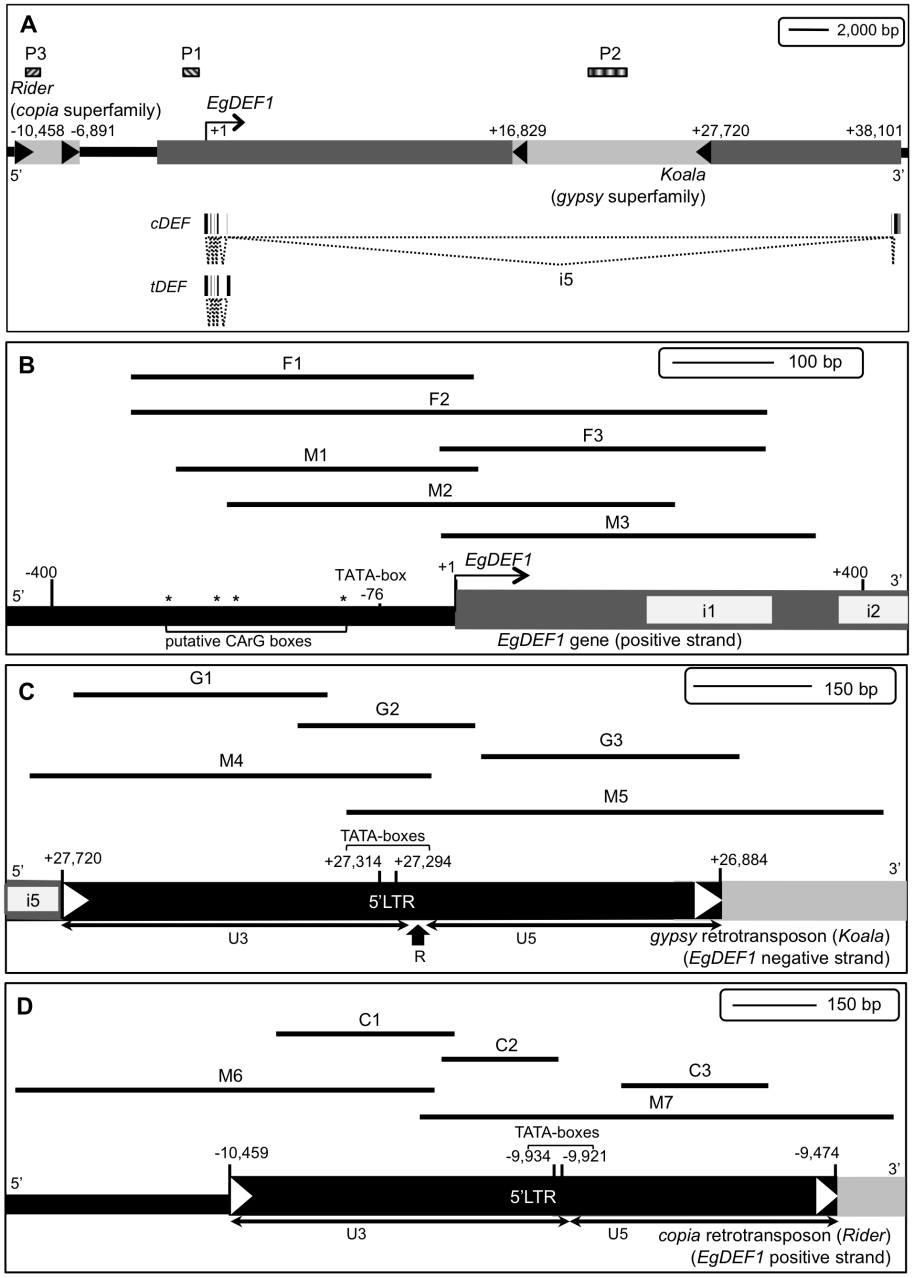
Structure of the *EgDEF1* gene region and localization of the sequences targeted in this study. A: Structure of the genomic region. Nucleotide positions are indicated with respect to the first coding base of the *EgDEF1* gene (+1 position). Dark grey box: *EgDEF1* gene; arrow: sense of transcription. Light grey boxes: retrotransposons; black arrowheads: LTRs. P1, P2 and P3: probes used in Southern blot experiments. The respective structures of the *cDEF* full-length transcript and of the alternative *tDEF* transcript are represented below the *EgDEF1* gene structure. Localization of the amplicons used in the DNA methylation analyses of the *EgDEF1* gene (B), the *Koala* retrotransposon (C) and the *Rider* retrotransposon (D). Fragments F1-F3, G1-G3 and C1-C3 were amplified from bisulfite-treated DNA, whereas fragments M1-M7 were amplified from McrBC-digested DNA. The complete list of primers is provided in Table S4. Black boxes delimited by white arrowheads: 5′LTRs; white boxes embedded in grey background: *EgDEF1* introns (numbered); the respective locations of the hypothetical TATA-boxes and CArG boxes (asterisks) are indicated.

Seven micrograms of oil palm genomic DNA extracts were digested in parallel by either EcoRI, HindIII or BamHI restriction enzymes (Promega) according to the manufacturer's instructions. The eletrophoretic separation of digestion products was performed as described previously [3] before transfer on a GeneScreenPlus membrane (Perkin Elmer). DNA templates for probe synthesis were obtained through the PCR amplification of regions corresponding to a sequence located immediately upstream of the *EgDEF1* gene (P1 probe), or to an internal section of each retroelement under study (P2 probe for the *gypsy* element, P3 probe for the *copia* element). The respective locations of the probes are illustrated in Figure 1A, probe sizes and primer sequences are given in Table S3. After purification using the Qiaquick kit (Qiagen), 25 ng of PCR product were radiolabeled with [α-^32^P]dCTP using the Random Primer DNA Labeling System Kit (Invitrogen). Hybridizations were performed at 65°C overnight in a mix of 100 μl PerfectHybrid Plus buffer (Sigma) plus 0.4 μl herring sperm DNA (Promega) per cm^2^ of membrane surface. Filters were then briefly washed at room temperature in 2× SSC, 0.1% (w/v) SDS, then at 65°C for 20 min in 2× SSC, 0.1% (w/v) SDS then at 65°C for 20 min in 1× SSC, 0.1% (w/v) SDS, before scanning the blots on a Typhoon 8600 Imager System (Amersham).

### Identification of *EgDEF1* transcripts by Rapid Amplification of cDNA Ends (RACE)

Transcripts originating from the *EgDEF1* gene were amplified by 3′-RACE using the SMART™ RACE cDNA Amplification kit and the Advantage 2 Polymerase (both from Clontech) using 3tsDEF-1 then 3tsDEF-1n as the gene-specific primer (Table S2). RACE products were then separated by agarose gel electrophoresis before purification using the Qiaquick PCR Purification Kit (Qiagen). Three microliters from the final 30 μL eluate were ligated overnight at 4°C into the pGEM-T Easy cloning vector (Promega). The transformation of thermocompetent JM109 cells (Promega) and plating was performed as recommended by the supplier. Five to ten positive colonies were selected for plasmid isolation with the Qiaprep Spin Miniprep Kit (Qiagen) and insert sequencing (Eurofins MWG Operon, Germany). Amplifications performed independently on normal and *mantled* oil palm inflorescences from genotypes FC166 and FC2317 (Table S1) yielded identical results.

### Bisulfite sequencing

The bisulfite conversion of cytosines was performed on 500 ng of purified DNA extract using the EZ Methylation Gold kit (Zymo Research). Only reactions with a C-T conversion rate higher than 98% (as determined by the sequencing of 10 amplicons obtained from the conversion of an unmethylated standard) were used in further analyses. Bisulfite-converted DNA was then used for the PCR amplification of selected target regions (Figure 1B–1D). The first round of amplification was performed on 2 μL of bisulfite-converted DNA using 10 pmol of each primer in a final volume of 50 μL according to the Advantage 2 Polymerase Kit (Clontech) protocol. We used a “touch-down” programme of 5 cycles (94°C 30 s, 60°C 30 s, 72°C 30 s) then 25 cycles (94°C 30 s, 55°C 30 s, 72°C 30 s). Then 0.5 μL of the product from this first round was used for a second round of amplification with nested or semi-nested primer combinations using GoTaq Polymerase (Promega) and a programme of 30 cycles (94°C 30 s, 55°C 30 s, 72°C 1 min). The complete list of bisulfite-specific primers is displayed in Table S4. The separation of amplification products, their purification, cloning and the selection and sequencing of positive inserts were performed as previously indicated. A minimum of 15 sequenced amplicons per primer pair and per sample was analyzed using the CyMATE software platform (http://www.cymate.org/) [23].

### McrBC-PCR

The protocol was adapted from [24] and optimized for oil palm genomic DNA. One microgram of genomic DNA was digested overnight at 37°C by 30 U of the McrBC enzyme (New England Biolabs) in a final volume of 40 μL. Controls containing no enzyme (“minus” tubes) were incubated alongside the digests (“plus” tubes). After heat-inactivating the enzyme (20 min at 65°C), the quality of DNA digestion was assessed by running 5 μl from each tube on a 1% agarose gel. Amplification was conducted on 1 μL from each tube using 2×10 pmol of each primer combination and the GoTaq Polymerase (Promega). The programme consisted in 30 cycles of (94°C 30 s, 60°C 60 s, 72°C 3 min). The list of the primers is given in Table S4 and their respective targets are shown in Figure 1B–1D. The presence or absence of the expected amplification product in digested *vs.* control was scored on an ethidium bromide-stained 1% agarose gel and the corresponding band was sequenced.

### cDNA synthesis and real-time quantitative PCR (rt-qPCR)

One microgram of total RNA extract was reverse-transcribed using the ImProm II kit (Promega) according to the manufacturer's instructions. In order to minimize artifacts caused by variations in RT yields, three reactions were performed from each RNA extract and pooled before being used as template in the subsequent rt-qPCR. Primer pairs were selected using the LightCycler Probe Design programme (Roche Applied Science) (Table S5). The Efficiency of each pair was assessed by amplifying serially diluted cDNA solutions: 2 μL of each dilution were added to the amplification mix composed of 1.5 μL of each primer at a 2 μM concentration and 5 μL of LightCycler 480 SYBR Green I Master mix (Roche Applied Science). Amplification was carried in a LightCycler 480 System (Roche Applied Science) using the following programme: 10 min at 95°C, then 45 cycles of 45 s at 95°C and 1 min at 70°C. Three independent amplifications (technical replicates) were performed to assess the reproducibility of results. The presence of contaminants was checked by performing a « no RT » negative control for each RNA sample and a « no template » negative control for each primer pair. After completion of the run, the Efficiency (E) was inferred from the linear regression of Crossing Point (Cp) values versus cDNA concentrations using the formula: E = 10^−1/slope^. Primer specificity was assessed through melting curves analysis and the sequencing of the amplification products. Transcript quantitation was then performed using the optimal template concentration determined previously, under identical amplification conditions. The fold-change in relative expression (RE) with respect to the normal inflorescence at stage 0 (used as calibrator) was calculated for each transcript with the formula [25]:
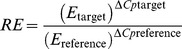
where ΔCp is the difference in Cp between the calibrator and the sample for either the target or the reference transcript (in this case the housekeeping gene *EgEF1α1*, accession AY550990.1).

In order to directly compare the respective accumulations of each transcript within the inflorescences, absolute quantitation was performed. To achieve this, we established calibration curves for each transcript using triplicated serial dilutions of the corresponding purified PCR product as the template in order to span the Cp range obtained previously in our material. The equations of the resulting linear regressions of Log [Copy number] vs. Cp (Figure S1) were then used to estimate the copy number of each transcript and calculate the following ratio:
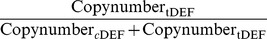
which was finally normalized across developmental stages with respect to the slight variations in *EgEF1α1* copy number.

### Statistical analysis

In order to identify phenotype-associated differences, the calculated methylation percentages and RE values were compared between normal and *mantled* samples using Student's two-tailed t-test. Data were compared between inflorescences of the same sex and developmental stage. For a given target sequence, methylation percentages were compared between phenotypes both individually for each site class (*i.e.* CG, CHG and CHH sites) and as an average.

## Results

### Isolation of the *EgDEF1* gene sequence and structure of the genomic region

**Figure 2:**
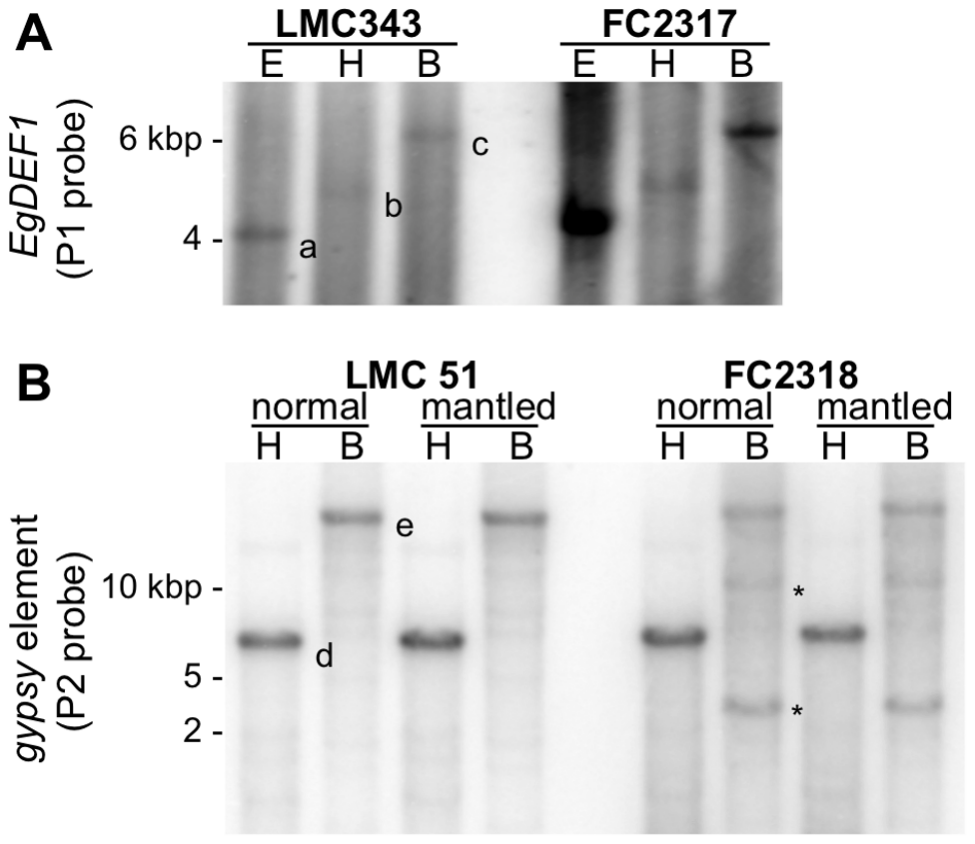
Determination of copy number by Southern blot. A: *EgDEF1* gene; B: *gypsy* retroelement. One L2T-related genotype (left: LMC343 or LMC51) is compared to one non-L2T-related genotype (right: FC2317 or FC2318). Lowercase letters signal the hybridizing bands that are predicted by the *in silico* digestion of the Eg133H20 BAC sequence with the appropriate restriction enzyme (a: 4,069 bp EcoRI fragment; b: 4,841 bp HindIII fragment; c: 6,251 BamHI fragment; d: 6,138 bp HindIII fragment; e: 13,132 bp BamHI fragment), asterisks indicate supernumerary bands.

The screening of a BAC library generated from a seed-derived L2T oil palm with *EgDEF1*'*s* putative promoter allowed the isolation of the Eg133H20 clone (accession number KF142646). The start of the *EgDEF1* coding sequence is localized near the middle of this 81.5 kbp genomic fragment from which the 3′ extremity of the gene (corresponding to exons 6 and 7) is missing, due to the presence of a massive intron 5 (36.3 kbp). Chromosome walking in 3′ orientation from the ATG confirmed both the structure and sequence of the *EgDEF1* gene. More walking in both 5′ and 3′ orientation using as reference the 3′ extremity of the *EgDEF1* cDNA enabled the recovery of the missing exons, the 3′end of the 5^th^ intron matching perfectly the Eg133H20 BAC clone border. The Southern hybridization pattern obtained using the P1 probe confirmed the occurrence of a single copy of the *EgDEF1* gene in both L2T-related (LMC343) and non-L2T-related (FC2317) oil palm genotypes (Figure 2A). The recent publication of the complete genome sequence of the oil palm [26] has allowed us to confirm the structure of the *EgDEF1* gene, which near-perfectly matches a portion of scaffold 00322 (BLASTN E value  = 0, identity 98–99%, score 5.373×10^4^ bits). The reassembled genomic sequence of the *EgDEF1* gene has been deposited in GenBank (accession number KF142645) and the gene structure is depicted in Figure 1A.

Further annotation of the BAC clone sequence showed that the genomic region surrounding the *EgDEF1* gene contains several sequences with similarities to Transposable Elements. Among these, two TEs raised our interest because of their proximity to the gene and the possibility that they could impact its regulation. One of these elements is 10.9 kbp long and is embedded within the 5^th^ intron of *EgDEF1*, in reverse orientation with respect to the gene. Its 5′ and 3′ LTRs are 837 and 815 bp long, respectively, and sequence similarity search through BLASTX showed that it is most similar to putative members of the *Ty3*/*gypsy* retrotransposons family detected in the genomes of the common bean (*Phaseolus vulgaris*), *Arabidopsis thaliana*, *Silene latifolia* (E value  = 0 for all three of them), grapevine (*Vitis vinifera*) (4e-172) and rice (*Oryza sativa*) (6e-163). This element, like 47% of the repeated elements detected in the oil palm genome, has no similarity to a previously characterized TE family [27]. A second element of interest, located upstream from *EgDEF1* and in the same orientation as the gene, is a much smaller retroelement (3.6 kbp) bounded by LTRs measuring 986 (5′) and 1005 bp (3′) respectively. The strongest BLASTX hits include putative *Ty1*/*copia* sequences detected in rice (E value 2e-61) and grapevine (1e-51) as well as the *Rider* retroelement of tomato (*Solanum lycopersicum*) (2e-53) and a member of the *Tnt1* retrotransposon family of tobacco (*Nicotiana tabacum*) (2e-52). Southern blots using the P2 probe (Figure 2B) reveal a banding pattern in accordance with a single “perfect” copy of the *gypsy* element inserted in the oil palm genome, and 3–4 more distantly related copies producing weaker hybridization signals. In the non-L2T-related FC2318 genotype, the occurrence of a supplementary BamHI restriction site in one of the two alleles of the gene is the most likely explanation for the decreased intensity of the e band and the parallel emergence of two supernumerary bands (approx. 10 kbp and 2,500 bp, respectively) resulting from its digestion. For both LMC51 (L2T-related) and FC2318 (non-L2T-related) genotypes, hybridization patterns generated by probes P1 and P2 are consistent with the occurrence of this particular *gypsy* insertion in both alleles of the *EgDEF1* gene. The same filter was stripped and re-hybridized with the P3 probe but the result, while suggesting a large number of hybridizing signals, was found inconclusive as to the copy number of the *copia* element (not shown). A repetition of this experiment yielded identical results. For both retrotransposons, there was no visible amplification of copy number in *mantled* oil palms when compared to normal ones. The comparison with the oil palm genome sequence later confirmed the occurrence of the *gypsy* insertion within the intron 5 of *EgDEF1* (E value  = 0, identity 99%, score 1.928×10^4^ bits), as well as the presence of shorter, partially matching sequences in other genomic locations. As for the *copia* retroelement, we found over 50 imperfect copies scattered throughout the genome (E value  = 0, maximum score 3029 bits). Since the sequenced genome belongs to a *pisifera* individual [26] which relatedness to our *tenera* (*dura* x *pisifera*) hybrids is unknown, we can only assume that this particular *copia* insertion is variable among oil palm genomes.

According to the convention suggested by Wicker *et al.* [27], we named these retroelements RLG_*Koala*_Eg133H20-1 (*gypsy* element) and RLC_*Rider*_ Eg133H20-1 (*copia* element), and they are thereafter refered to as *Koala* and *Rider* in the present paper. Both TE sequences have been deposited in GenBank under accession numbers KF142647 and KF142648, respectively.

### Methylation analyses

**Figure 3:**
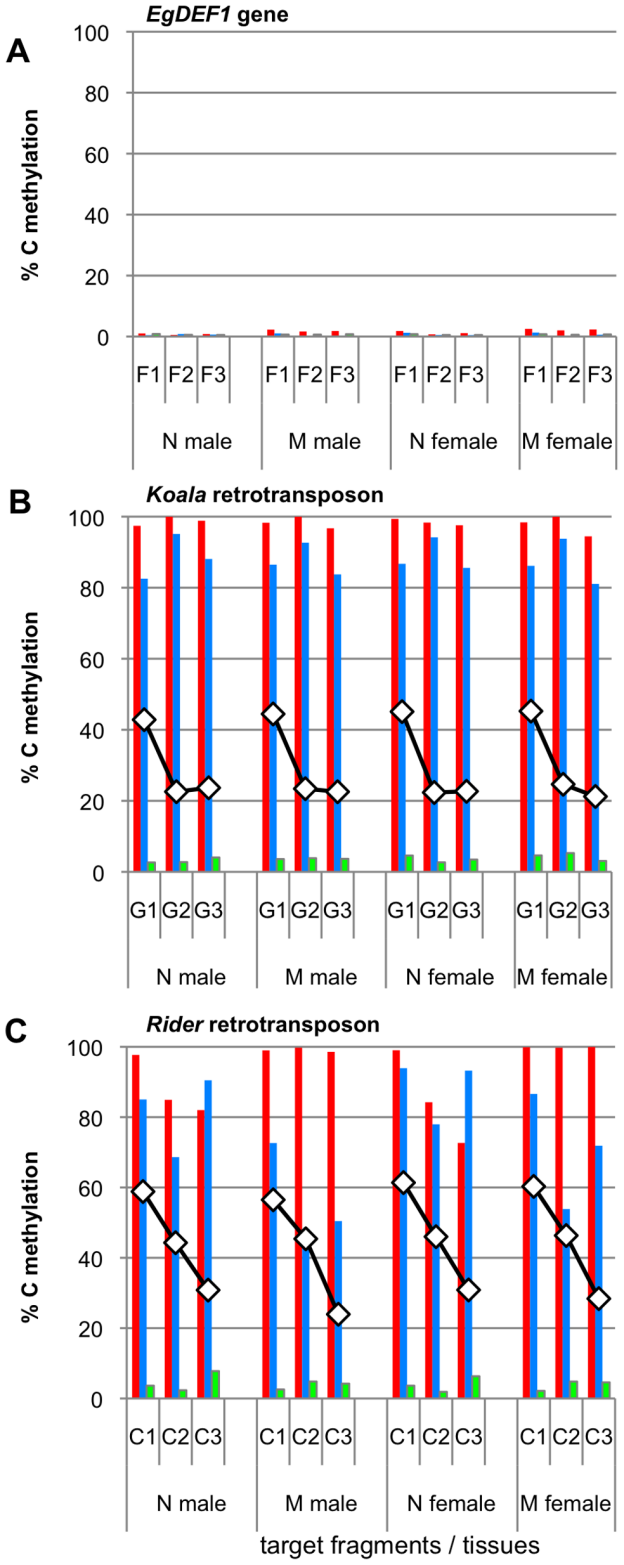
Site-specific analysis of DNA methylation by bisulfite sequencing. A: *EgDEF1* gene; B: *Koala* retrotransposon; C: *Rider* retrotransposon. The percentage of methylated CG, CHG and CHH sites (where H is A, T or C) within each region investigated is represented as red, blue and green bars respectively. The average methylation rate throughout each sequence is represented as diamonds (because of the scale, these percentages could not be represented on the *EgDEF1* graph). N, M: inflorescence sampled on a clonal oil palm of either normal or *mantled* floral phenotype, respectively. The localization of PCR fragments on their respective target sequence is as indicated in Figure 1.

**Figure 4:**
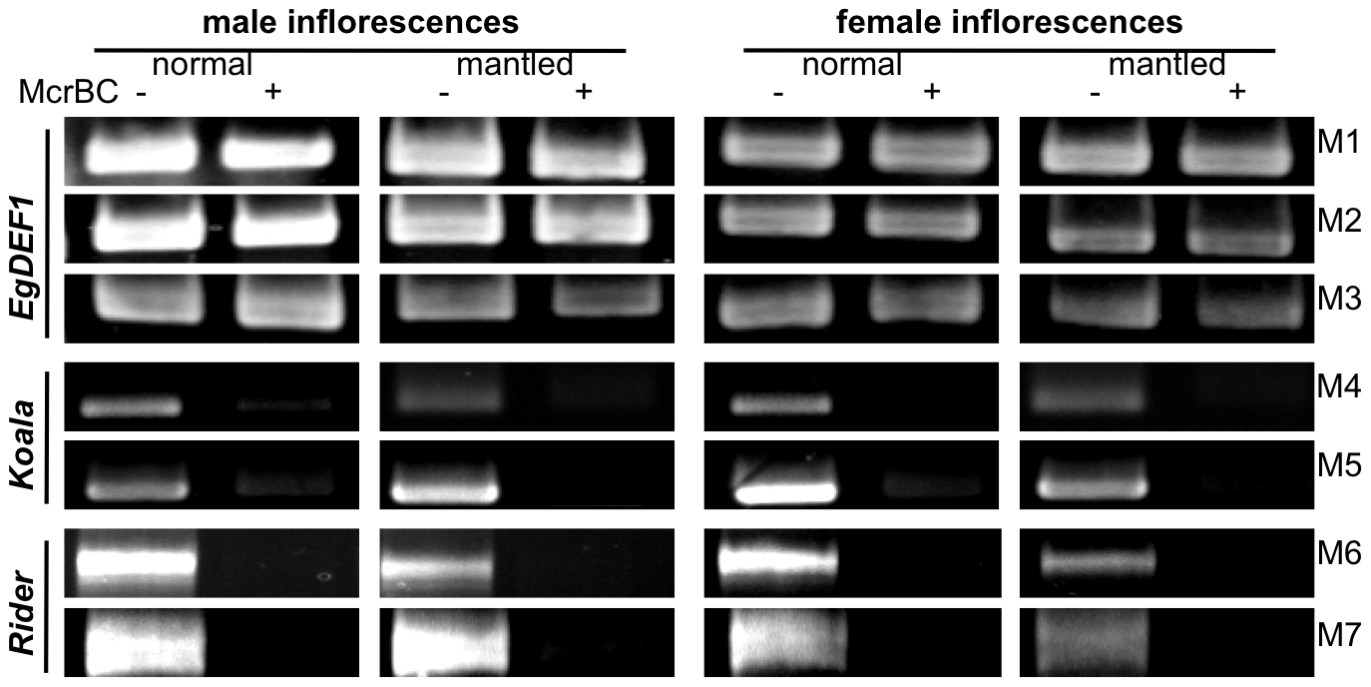
McrBC-PCR analysis of DNA methylation within the target regions. Plus and minus signs correspond to amplifications performed from DNA samples incubated in the presence or in the absence of the McrBC enzyme, respectively. The localization of the M1 to M7 PCR fragments is illustrated in Figure 1.

Bisulfite sequencing experiments targeting the region of the *EgDEF1* gene spanning *ca.* 300 bp of proximal promoter sequence to the 5′ extremity of exon 4 revealed a very low percentage of cytosine methylation. As illustrated by Figure 3A, the average methylation rates within the different amplicons studied ranged from 0.53 to 1.05% depending on the sex and phenotype of the inflorescence. Furthermore, by performing a position-specific analysis of methylation we could not detect any local accumulation of methylation in the promoter region covered by fragments F1 and F2. This region includes motifs involved in the transcriptional regulation of MADS-box genes such as the four putative CArG boxes (at positions -106, -211, -229 and -278, respectively) and the putative TATA-box (position -76) (Figure 1B and Figure S2). However, such methylation rates do not allow a reliable discrimination between methylated Cs and unmethylated, unconverted Cs since the latter have a maximal frequency of 2% under our experimental conditions. The results from the McrBC-PCR analysis are consistent with the absence or near-absence of cytosine methylation within this region (Figure 4).

The 5′LTR sequence of the *Koala* retroelement displays strong methylation throughout all three PCR fragments examined, irrespective of inflorescence sex or phenotype. This is supported by both bisulfite (Figure 3B and Figure S3) and McrBC-PCR data (Figure 4). Overall, 94.5 to 100% of CG sites and 81 to 95% of CHG sites are methylated, contrasting with a low level of CHH methylation (2.6 to 5%). The differences in the average methylation of amplicon G1 (44.4%) *vs.* G2 and G3 (23.2 and 23.5%, respectively) mirror the differences in site composition of these sequences (Figure S3 and Table S6).

The amplification of G3 led to the identification of two alleles carrying distinct Single Nucleotide Polymorphisms (SNPs) resulting in the occurrence of two supplementary CG sites in allele G3-a2 compared to G3-a1 (Table S6). However, the distribution of methylated sites across the G3 sequence is similar in both alleles (Table S7 and Figure S3 C–D), a result that is further confirmed by McrBC-PCR on the M5 fragment (Table S8 and Figure 4). No statistically significant changes in DNA methylation rates are found between normal and *mantled* flower phenotypes either at the site or whole amplicon level (Table 1).

**Table 1:** Student t-test comparison of mean methylation rates between normal (N) and *mantled* (M) inflorescences.

Tissue	Methylation	p-value					
		*Koala*			*Rider*		
		G1	G2	G3	C1	C2	C3
**male inflorescences**	CG	0.65	n.a.	0.44	4.7×10^−2^ *	2.2×10^−12^ ***	3.1×10^−4^ ***
	CHG	0.10	0.22	9.2×10^−2^	1.1×10^−4^ ***	4.5×10^−7^ ***	4.5×10^−12^ ***
	CHH	0.15	0.14	0.71	3.8×10^−2^ *	0.15	9.2×10^−2^
	average	0.11	0.25	0.18	3.8×10^−3^ ***	0.39	4.9×10^−3^ **
	**n normal**	**43**	**30**	**36**	**57**	**28**	**22**
	**n ** ***mantled***	**26**	**29**	**31**	**48**	**29**	**28**
**female inflorescences**	CG	0.25	0.43	0.23	2.8×10^−2^ *	7.2×10^−12^ ***	1.7×10^−10^ ***
	CHG	0.89	0.75	0.49	9.7×10^−4^ ***	5.2×10^−14^ ***	5.6×10^−10^ ***
	CHH	0.81	5.4×10^−2^	0.95	4.5×10^−2^ *	9.2×10^−4^ ***	0.14
	average	0.74	7.9×10^−2^	0.30	0.27	0.75	1.1×10^−2^ *
	**n normal**	**35**	**31**	**35**	**28**	**45**	**30**
	**n ** ***mantled***	**55**	**20**	**36**	**37**	**27**	**28**

n normal and n *mantled* are the number of individually cloned amplicons analyzed for normal and *mantled* inflorescence samples, respectively.

n.a.: not applicable; *: p<0.05; **: p<0.01; ***: p<0.001; all other values are not significant.

Results of the methylation analysis for the *Rider* retroelement also demonstrate that its 5′LTR is highly methylated (Figure 3C and Figure 4) although the distribution of the methylated Cs is distinct from that of *Koala*. First, the average methylation rate of the amplicons decreases steadily from the 5′ to the 3′ extremity of the LTR, concurrently with the decrease in both C content and CG frequency from C1 to C3 (respectively 105 cytosines and 41% of CG sites *vs*. 43 and 11.6%) (Figure 3C, Table S6 and Figure S4). Moreover, statistically significant differences are observed in relationship with the phenotype within all three amplicons, which show higher CG methylation and lower CHG methylation in *mantled* inflorescences of both sexes (Figure 3C and Table 1), a conclusion that is supported by the position-specific analysis (Figure S4).

### Analysis of retrotransposons expression

The production of transcripts by *Koala* and *Rider* elements was assessed by rt-qPCR. The accumulation of RNA produced by *Koala*, as detected with the RT1-QF5/QR5 primer pair, increases throughout both male and female inflorescence development (Figure S5 A and B, top panels). However, the very elevated Cp values obtained indicate that this transcript production cannot be distinguished from the background noise and our results are highly variable among technical replicates. As a result, the comparison of Relative Expression (RE) between normal and mantled inflorescences yielded non-significant p-values in most cases. Two other primer pairs were tested (Table S5 and Figure S6) and gave similar results (not shown).

By contrast, the expression of *Rider* was readily detected using the RT2-QF1/QR1 primer pair (Figure S5 A and B, bottom panels) and it was found to be mostly stable throughout development of both normal and *mantled* inflorescences. Moreover, we detected statistically significant differences in RE in relationship with the floral phenotype, with male and female *mantled* inflorescences displaying higher expression of *Rider* at most developmental stages compared to their normal counterparts. Virtually identical results were obtained with the RT2-QF3/QR3 pair (not shown).

### Analysis of *EgDEF1* gene expression

The discovery of *EgDEF1* gene structure raises the question of possible dysfunctions of either transcription or pre-mRNA processing mechanisms resulting from the extreme size of intron 5. Indeed, our 3′-RACE-PCR experiments show that at least two distinct transcripts are produced from the *EgDEF1* gene. As illustrated by Figures 1A and S7, in addition to the 979 nucleotides-long full-length transcript (hereafter named *cDEF*) we detect a slightly shorter polyadenylated molecule (845 nucl). This alternate transcript contains exons 1 to 4 and includes the 5′ extremity of intron 5 with two small deletions (5 and 13 nucl respectively) inducing two successive frameshifts. Because of the truncated coding sequence of the alternate transcript, we named it *tDEF* (accession number KF142649). A BLASTN search in the available oil palm inflorescence transcrip datasets [26], [28] confirmed the occurrence of several shorter isoforms similar to *tDEF*.

**Figure 5:**
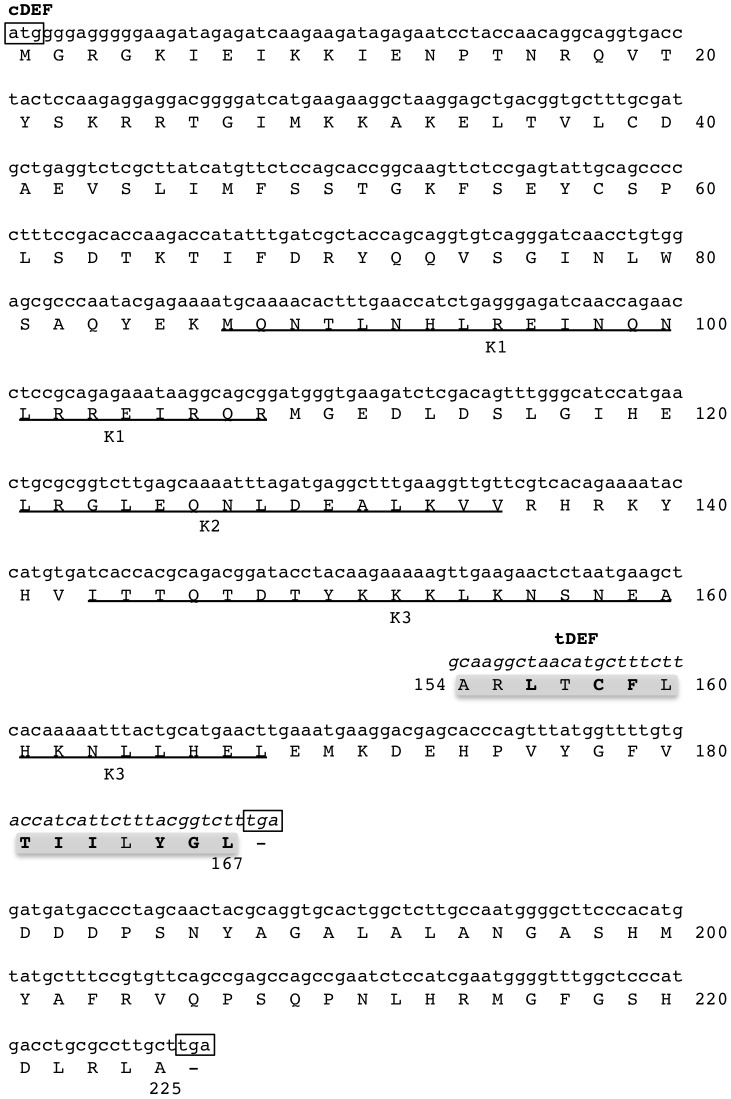
*In silico* translation of the *cDEF* and *tDEF* transcripts. Start and stop codons are boxed. The peptidic sequence hypothetically produced by the full-lenght transcript (*cDEF*) of the *EgDEF1* gene has been inferred from the nucleotidic sequence using Translate (http://web.expasy.org/translate/). Only the portion of the *tDEF* transcript sequence that differs from *cDEF* is represented in the figure (italics), and the resulting peptidic sequence is shaded in grey. Residues of the alternate peptide with modified biochemical properties with respect to their counterpart in the native peptide are in bold. The three motifs forming the K box domain are underlined.

In the aim of evaluating the potential consequences of structural alterations in *tDEF* with respect to *cDEF*, we compared the putative peptidic sequences derived from each transcript (Figure 5). The hypothetical product of *tDEF* is 58 residues shorter than the complete peptide and therefore lacks the C-terminal domain. Moreover, its K domain is modified in the K3 motif since 9 of the 14 aminoacids originating from the partial read-through of intron 5 correspond to altered biochemical properties with respect to the functional peptide.

**Figure 6:**
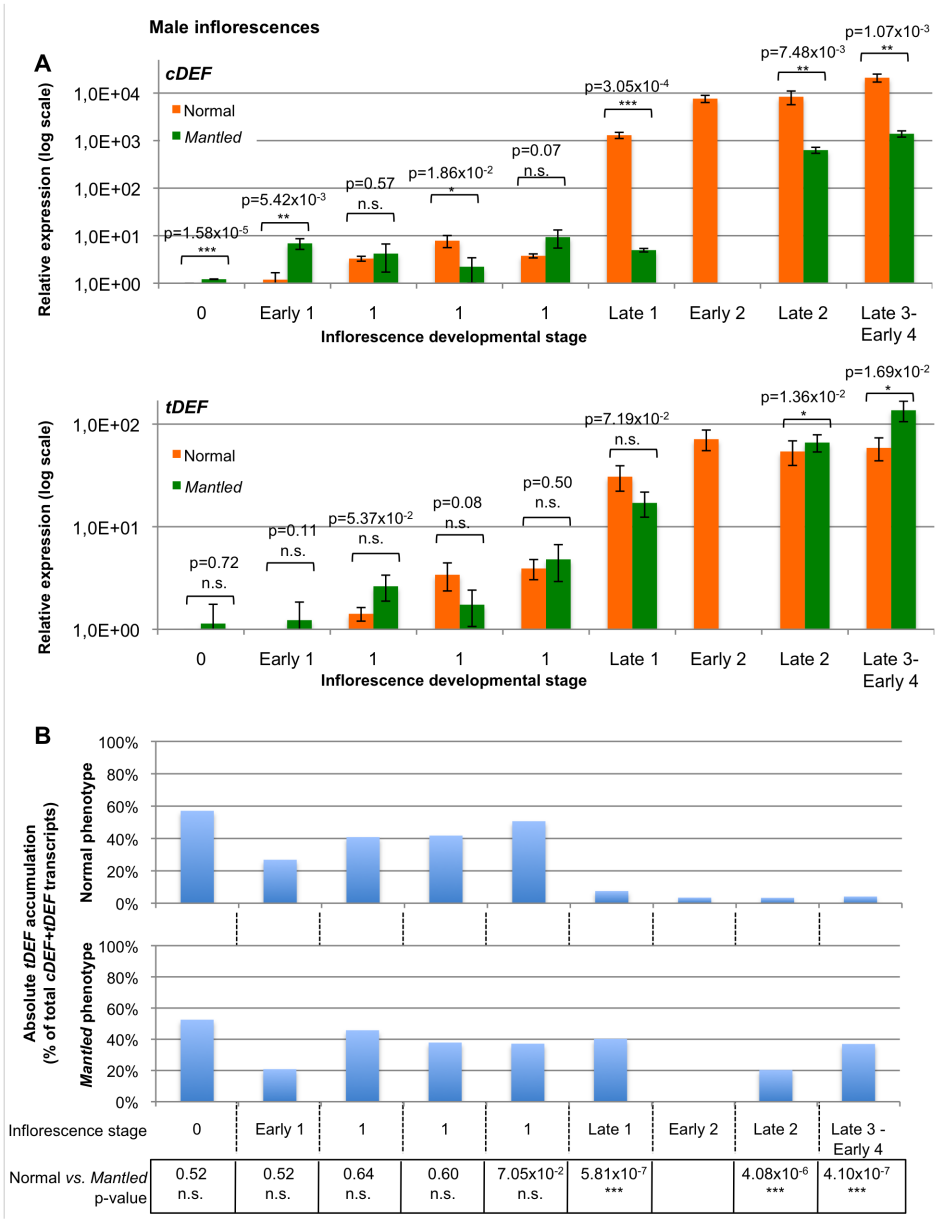
Real-time qPCR quantitation of *cDEF* and *tDEF* transcript accumulation in developing male inflorescences. Inflorescences were sampled from adult palms of either normal (orange) or *mantled* (green) phenotype. Developmental stages are numbered from 0 to 4 according to Adam et al (2007). A: Relative Expression (RE) values for *cDEF* (top) and *tDEF* (bottom) are calibrated against stage 0 normal inflorescences and displayed as means of three technical replicates ± Standart Deviation. P-values obtained through the comparison of the REs between normal and *mantled* inflorescences with Student's two-tailed t-test are indicated (n.s.: not significant; *: p<0.05; **: p<0.01; ***: p<0.001). B: Absolute *tDEF* transcript accumulation in normal (top) and *mantled* (bottom) inflorescences represented as a percentage of *tDEF* copy number over the sum of *cDEF* and *tDEF* copy numbers within each sample, normalized with respect to the reference gene. The p-values obtained through the stage-by-stage comparison of *tDEF*/*cDEF* ratios between comparable normal/*mantled* inflorescence pairs are displayed below the graphs.

In order to understand the dynamics of *EgDEF1* expression in the developing oil palm inflorescence, we designed rt-qPCR primers targeting specifically the *cDEF* or the *tDEF* transcript (Figure S7 and Table S5). Both transcripts were detected in samples from all developmental stages, including stage 0. In the normal male inflorescence (Figure 6A, top panel) the accumulation of the *cDEF* transcript undergoes a sharp increase with respect to its initial level at stage 0 and this effect gradually recedes throughout developmental stages: over 1,000-fold between Early and Late stage 1, then nearly 6-fold between Late stage 1 and Early stage 2 and 1.5-fold between late stage 2 and stage 3. In the *mantled* male inflorescence, the increase appears to be delayed and more modest in magnitude: about 130-fold between Late stage 1 and Late stage 2, then over 2-fold between Late stage 2 and Late stage 3 - Early stage 4. For each comparable developmental stage, we found statistically significant differences in *cDEF* accumulation between normal and *mantled* male inflorescences, with higher expression of *cDEF* in normal tissues.

As for the *tDEF* transcript (Figure 6A, bottom panel), its upregulation occurs earlier than for *cDEF* in the normal male inflorescences since its accumulation is increased 8-fold in the course of stage 1. However this increase slows down rapidly: 2-fold increase between Late stage 1 and Early stage 2 then the expression of *tDEF* reaches a plateau. By contrast, in the corresponding variant inflorescence the increase in *tDEF* accumulation is sustained until the later stages of inflorescence development: 4-fold between both the two latest Stage 1 inflorescences and Late stage 1 and Late stage 2, then 2-fold between Late stage 2 and Late stage 3 - Early stage 4. Differences in *tDEF* expression between normal and *mantled* male inflorescences were statistically significant for the last two developmental stages studied, with higher accumulation of *tDEF* observed in *mantled* inflorescences.

We tried to determine whether the expression level of *tDEF*, compared to that of *cDEF*, was sufficient to support the hypothesis of a competition between both transcripts. To achieve this, we performed absolute qPCR quantitation of each transcript within each inflorescence and used the resulting transcript copy numbers to express the accumulation of *tDEF* as a percentage of the *cDEF* + *tDEF* total. As show in Figure 6B, during the earliest stages (0-1) of both normal and *mantled* male inflorescence development the truncated transcript accounts for up to half of the total *EgDEF1* transcript accumulation, with no statistically significant difference in these proportions between the two floral phenotypes. In the true-to-type inflorescence the proportion of *tDEF* drops abruptly from 48% to 8.5% at Late stage 1, then stabilizes around 4–5% throughout the following developmental stages. By contrast, in the developing abnormal male inflorescence the percentage of *tDEF* remains mostly stable (37–40% of *EgDEF1* transcripts) with the exception of Late Stage 2 (20%). The differences in the *tDEF*-to-*cDEF* absolute ratio between normal and *mantled* male inflorescences were found to be statistically significant for all comparable inflorescence stages from Late stage 1 onwards.

We obtained similar results in female inflorescences (Figure S8A), the upregulation of both *EgDEF1* transcripts being both less important and more variable than in their male counterparts. Nevertheless, the respective proportions of *cDEF* and *tDEF* (Figure S8B) are significantly different between the two floral phenotypes from Late Stage 2 – Early Stage 3 onwards, with *tDEF* representing roughly 7% of total *EgDEF1* transcripts in Stage 4 normal female inflorescences compared to 18–28% in the corresponding *mantled* inflorescences.

## Discussion

The present work describes for the first time the structure of the *EgDEF1* gene, the oil palm ortholog of the B-class MADS-box genes *APETALA3* (*Arabidopsis thaliana*) and *DEFICIENS* (*Antirrhinum majus*). Very large introns (*i.e.* several kbs long) are commonly found in members of this gene superfamily [29]. The singularity of intron 5 in the *EgDEF1* gene resides in its extreme size of 36.3 kb, an enlargement which is partially attributable to the insertion of the 10.9 kbp *Koala* retrotransposon. The presence of intronic TE-derived sequences has been documented for several other large introns of MADS-box genes [30]–[32] and a survey of *AP3*-like genes in public databases showed that such remnants are detectable in most of them (Table S9). However, the intronic insertion of *Koala* is not, in itself, at the origin of the *mantled* phenotype. Indeed, we identified this insertion in both our BAC library and the recently published *pisifera* oil palm genome sequence [26], both originating from seed-derived palms that were therefore devoid of somaclonal variation.

Most TEs found in plant genomes are maintained in a constitutively repressed state through epigenetic silencing involving both a high level of DNA methylation at symmetrical sites (CG and CHG) and the formation of highly condensed heterochromatin [33], [34]. Moreover, several recent studies have shown that the complete de-repression of TE activity involves a massive decrease in LTR methylation (up to 80–90%) [33]–[36]. Both retrotransposons studied here display high methylation in both CG and CHG sequence contexts, with different consequences on their respective expression. We show that *Koala* does not display significant differences in either DNA methylation or expression between normal and *mantled* inflorescences. Taken together, these results suggest that this retroelement is efficiently silenced in our material and that it is not reactivated in the hypomethylated genome context of the *mantled* variation, making it unlikely that it can actively interfere with *EgDEF1* expression. Such a conclusion is further supported by ongoing experiments indicating that *Koala* is not transcriptionally or transpositionally reactivated in the course of *in vitro* micropropagation (T. Beulé, unpublished data). By contrast, in *mantled* inflorescences of both sexes the 5′LTR of *Rider* exhibits a combination of slightly increased CG methylation and strongly decreased CHG methylation compared to the corresponding normal inflorescences. These statistically significant changes are associated with higher transcript accumulation in *mantled* inflorescences at most developmental stages. This partial release from silencing in abnormal tissues could be the consequence of the decreased CHG methylation, since the imposition of this mark is functionally associated to heterochromatin formation [37]. Whether the expression of *Rider* interferes with that of *EgDEF1* in *mantled* inflorescences will have to be explored in future studies.

In parallel, after studying DNA methylation within the proximal promoter and first 400 bp of *EgDEF1* coding sequence we find that this sequence is essentially unmethylated in normal inflorescences. Also, no methylation change can be associated with either the *mantled* floral phenotype or with repressive epigenetic marks spreading from the two retrotransposons, whereas major differences in transcript accumulation are observed between normal and variant inflorescences. Globally we can conclude that, in the context of the somaclonal variation in oil palm, the regulation of *EgDEF1* expression does not depend on DNA methylation changes, nor is it directly affected by the genome-wide deficit in DNA methylation characterized previously in *mantled* tissues [2], [7].

With respect to previous results obtained through semi-quantitative RT-PCR by Adam *et al*. [38], we show that significant *EgDEF1* transcript accumulation can be detected at earlier stages of inflorescence development than previously thought and we identify an alternative, truncated transcript produced by the *EgDEF1* gene. According to current estimates, Alternative Splicing (AS) affects as much as 61.2% of intron-contaning genes in Arabidopsis [39]. A large number of MADS-box genes, including organ identity genes, undergo AS in a wide range of plant species [29], [40]–[48] and Jiao and Meyerowitz [49] have shown an increased frequency of AS events during floral organ differenciation in Arabidopsis. Our qPCR experiments demonstrated that the balance between the respective absolute accumulations of the functional (*cDEF*) and the truncated (*tDEF*) transcript is differentially regulated according to the floral phenotype. Remarkably, the stronger phenotype-dependent effect on the *tDEF*/*cDEF* ratio we observed in male inflorescences is consistent with the higher requirement of this tissue for the expression of genes controlling the formation of functional male organs, whereas only rudimentary and abortive stamens are found in female inflorescences [19]. This result is also in line with the greater severity of the *mantled* variation observed in clonal palms bearing abnormal male inflorescences. The fact that *cDEF* accounts for 60–63% (male) to 71–81% (female) of *EgDEF1* transcript accumulation throughout the stages of *mantled* inflorescences development during which stamens are formed (*vs.* 96 and 93% in the corresponding normal inflorescences, respectively) could result in a decreased production of the full-length peptide. Such a mechanism has been demonstrated in the case of the *FCA* gene of *Arabidopsis thaliana* [50]–[52] for which the amount of functional peptide synthesized is regulated through the balance between several alternative transcripts. A similar regulation of *EgDEF1* gene expression could potentially result in the formation of an insufficient amount of the DEF-containing ternary protein complex governing stamen formation in *mantled* inflorescences and, ultimately, in abnormal flower development.

Another possibility is that both transcripts are translated and that the truncated peptide competes with the native peptide for the generation of ternary complexes. In both APETALA3 and PISTILLATA proteins, the K3 motif is essential for both the formation of MADS complexes and the specification of organ identity, whereas the C domain could contribute to complex stabilization [53]–[56]. According to this scenario, the hypothetic tDEF peptide could generate non-functional complexes, leading to altered flower morphology through a dominant-negative effect [57]. The tDEF peptide will need to be isolated and quantified, and its binding properties will have to be compared to its full-length counterpart before this hypothesis can be validated.

What is the source of the AS event giving rise to the alternative *tDEF* transcript and how this phenomenon is altered in *mantled* inflorescences are still pending questions. Although the *Koala* element is distant from the borders of intron 5, we cannot exclude that the surrounding heterochromatin could interfere with the splicing of the host intron and contribute to the formation of *tDEF*. Two recently published articles [58], [59] have shown that intronic heterochromatin associated with TE insertions could influence pre-mRNA processing mechanisms, resulting in a modified balance between full-length and shorter transcript isoforms. The involvement of a similar process in the expression of *EgDEF1* and in the emergence of the *mantled* variation will have to be demonstrated. Nevertheless, this attractive hypothesis would be in accordance with an increasing number of publications demonstrating the occurrence of a crosstalk between epigenetic regulation processes and mRNA splicing in both animals [60]–[62] and plants [63]–[66].

While the present study does not allow us to establish direct causal relationships between DNA methylation, alternative transcript production and phenotypic plasticity, the next step will be to investigate the interactions between these phenomena. With this aim in mind, we have undertaken a high-throughput analysis of the transcriptome in developing oil palm inflorescences, which will allow us to determine whether the production of an alternative transcript by the *EgDEF1* gene is an isolated event or if this is part of a large-scale misregulation of mRNA processing mechanisms in *mantled* oil palm inflorescences.

## Supporting Information

Figure S1 **Calibration curves for the** ***EgEF1α1*** **,** ***cDEF*** **and** ***tDEF*** **transcripts.** y1, y2 and y3 are the equations of the Log [Copy number] vs. Cp curves obtained for the *EgEF1α1* (black diamonds), *cDEF* (white squares) and *tDEF* transcripts (grey triangles), respectively.
(PDF)

Figure S2 **Position-specific analysis of DNA methylation within the** ***EgDEF1*** **gene by bisulfite sequencing.** A-C: PCR fragments F1, F2, and F3. The methylation percentage at every cytosine position within the sequence is represented by black bars. The symbol below the horizontal axis corresponds to the sequence context of the corresponding C: CG is a red disc, CHG is a blue square, CHH is a green triangle. N, M: inflorescence sampled on a clonal oil palm of either normal or *mantled* floral phenotype, respectively. The localization of PCR fragments on their respective target sequence is as indicated in Figure 1. n is the number of individually cloned amplicons included in the study for each experimental condition.
(PDF)

Figure S3 **Position-specific analysis of DNA methylation within the** ***Koala*** **retrotransposon by bisulfite sequencing.** A–D: PCR fragments G1, G2 and G3 (allele 1 and 2). For legend see Figure S2.
(PDF)

Figure S4 **Position-specific analysis of DNA methylation within the** ***Rider*** **retrotransposon by bisulfite sequencing.** A–C: PCR fragments C1, C2 and C3. For legend see Figure S2.
(PDF)

Figure S5 **Real-time qPCR quantitation of transcripts produced respectively by the** ***Koala*** **and** ***Rider*** **retroelements.** In male (A) and female (B) inflorescence developmental series of normal (orange) or *mantled* (green) phenotype, we evaluated the expression of the *Koala* (top) and *Rider* (bottom) retroelements. For each TE, p-values obtained through the comparison of the REs between normal and *mantled* inflorescences with Student's two-tailed t-test are indicated (n.s.: not significant; *: p<0.05; **: p<0.01; ***: p<0.001).
(PDF)

Figure S6 **Localization of the rt-qPCR primers targeting the retrotransposons under study.** A: *Koala*; B: *Rider*. Target Site Duplications (TSD) appear in italics; LTRs are in bold. Sequences are displayed according to the 5′-3′orientation of their respective ORFs. The complete list of primers is available in Table S5.
(PDF)

Figure S7 **Alignment of two transcripts produced by the** ***EgDEF1*** **gene.** Genomic: genomic sequence (note that only the 5′ and 3′ extremities of intron 5 are represented on this figure; the missing part of the genomic sequence is replaced by a double slash symbol at each gap border). cDEF: full-length *EgDEF1* transcript; tDEF: truncated *EgDEF1* transcript (see manuscript for details). Start and Stop codons are in bold, 3′-UTR regions of each transcript are in italics. Exons are numbered from e1 to e7 and introns (shaded in grey) from i1 to i6. The sequences matching the rt-qPCR primers used to amplify each transcript (Table S5) are underlined.
(PDF)

Figure S8 **Real-time qPCR quantitation of** ***cDEF*** **and** ***tDEF*** **transcript accumulation in developing female inflorescences.** See Figure 6 for legend.
(PDF)

Table S1 Genetic origin of the plant material. Conventionally, crosses are given under the form “male parent x female parent”.
(PDF)

Table S2 List of primers used in genome walking and RACE experiments. Primer names beginning with “5” and “3” are primers designed for the amplification of sequences located respectively 5′ or 3′ relatively to their target region. Primer names ending with “n” are used in nested amplifications.
(PDF)

Table S3 Probes used in Southern blot experiments. The position of each probe relatively to the first coding base of the *EgDEF1* gene is indicated in Figure 1.
(PDF)

Table S4 List of primers used in DNA methylation analyses. Primers used to amplify the fragments depicted in Figure 1 are indicated. Primer names beginning with the letter “b” were used in bisulfite sequencing analyses, those beginning with “m” were used in McrBC-PCR analyses.
(PDF)

Table S5 List of primers used for rt-qPCR. Primer position on their respective target sequence is shown in Figures S6 and S7.
(PDF)

Table S6 Sequence characteristics of the amplification fragments analyzed through bisulfite sequencing. For the localization of each bisulfite-PCR fragment on the corresponding target region (*EgDEF1* gene or retrotransposons), see Figure 1. For the list of primers used to generate these fragments, see Table S5.
(PDF)

Table S7 Allele-specific analysis of DNA methylation within the G3 region of the *Koala* retrotransposon. n is the number of individually cloned amplicons analyzed and the average of methylation rates between both alleles is weighted accordingly.
(PDF)

Table S8 Sequence characteristics of the amplification fragments analyzed through McrBC-PCR. *McrBC half-sites are two RmC dinucleotides (where R is A or G and mC is a methylated cytosine) separated by 55 to 103 nucleotides in optimal conditions, the cut occurring randomly between them. For the localization of each McrBC-PCR fragment on the corresponding target region (*EgDEF1* gene or retrotransposons), see Figure 1. For the list of primers used to generate these fragments, see Table S5.
(PDF)

Table S9 Size of intron 5 in orthologs and putative orthologs of the *APETALA3* and *DEFICIENS* genes. The presence of TE-related sequences in the largest intron was assessed using CENSOR (http://www.girinst.org/censor/index.php).
(PDF)
