# Supplementary figures and images for: DNA Methylation and Expression of the EgDEF1 Gene and Neighboring Retrotransposons in mantled Somaclonal Variants of Oil Palm
Source: PLoS One. 2014 Mar 17;9(3):e91896. doi: 10.1371/journal.pone.0091896 (PMC3956824; doi:10.1371/journal.pone.0091896)

Figure S1

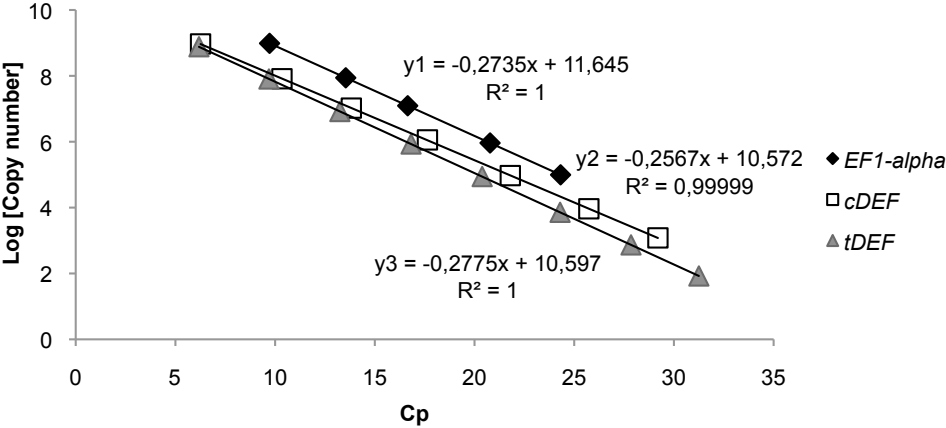

Supplement: Figure S1 — Calibration curves for the EgEF1α1 , cDEF and tDEF transcripts. y1, y2 and y3 are the equations of the Log [Copy number] vs. Cp curves obtained for the EgEF1α1 (black diamonds), cDEF (white squares) and tDEF transcripts (grey triangles), respectively. (PDF) [file pone.0091896.s001.pdf]

Figure S2

A F1 sequence

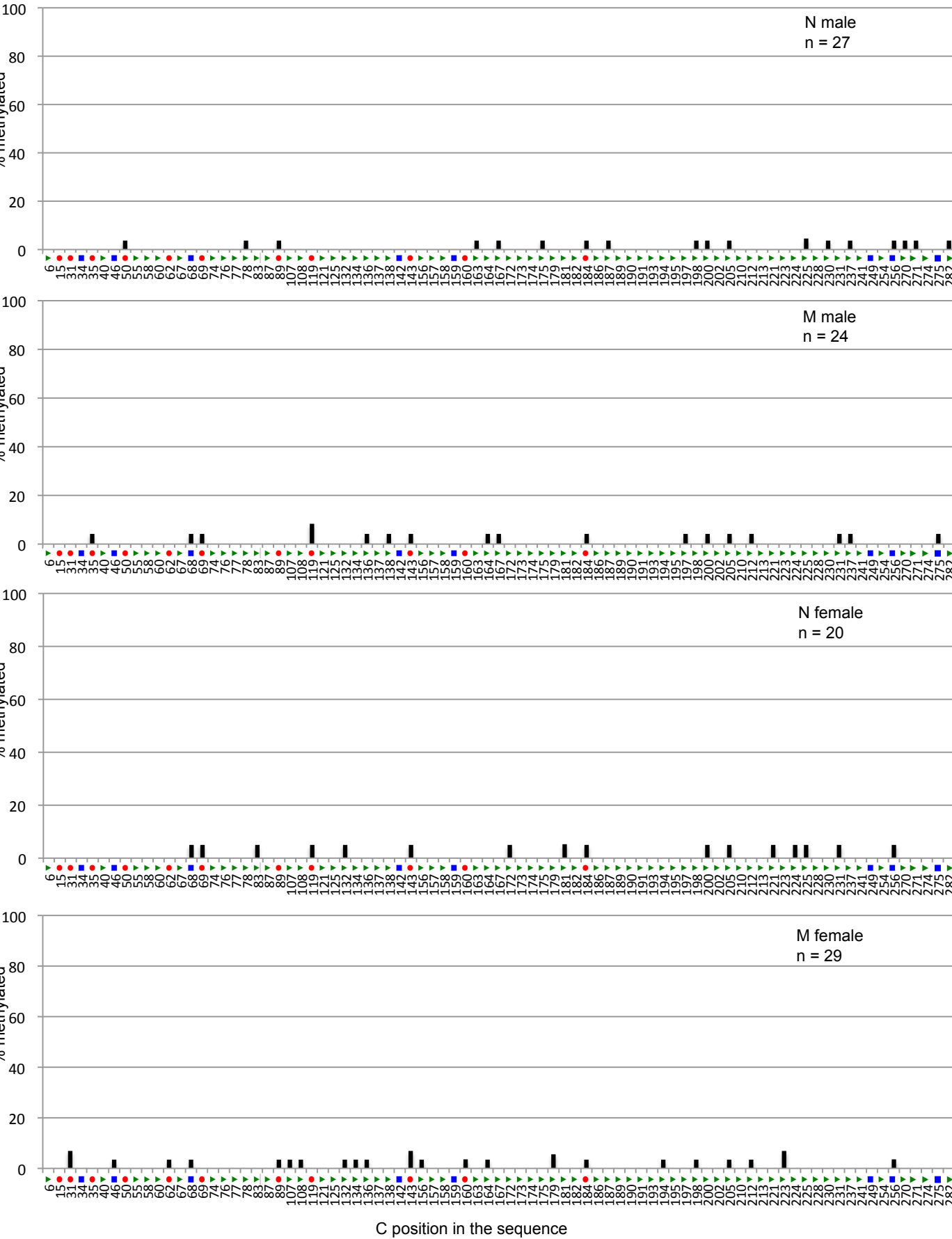

Figure S2

B

F2 sequence

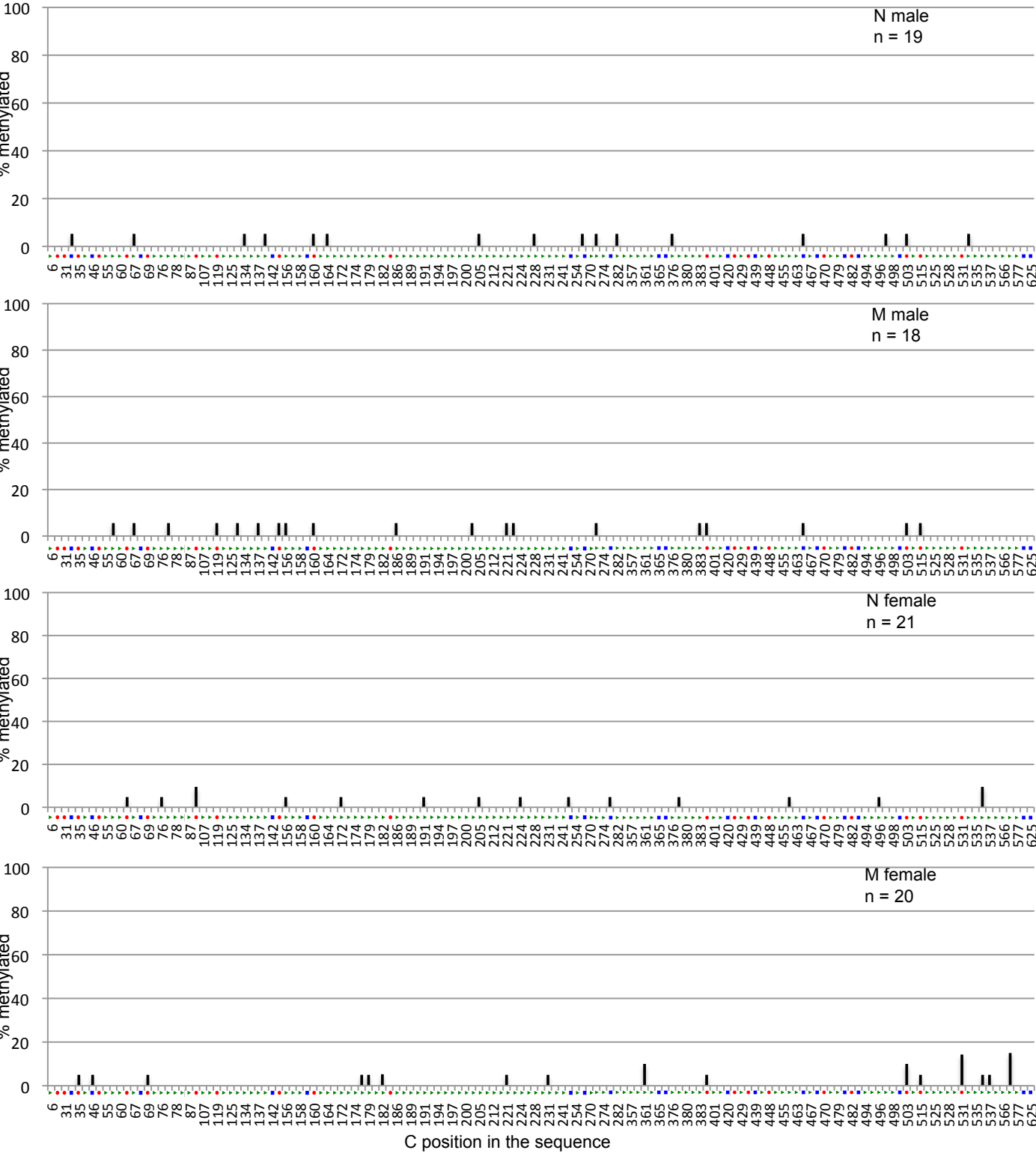

Figure S2

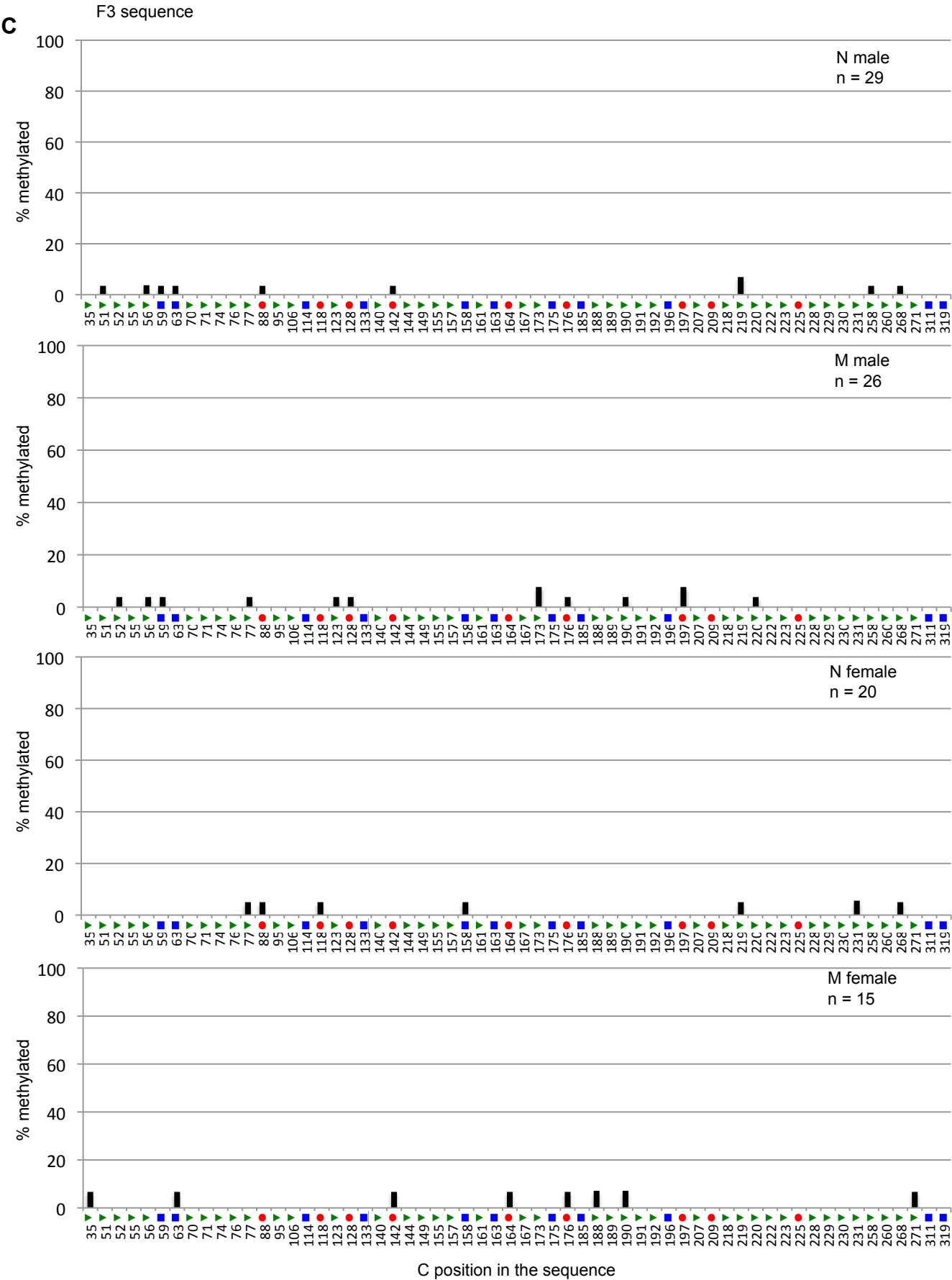

Supplement: Figure S2 — Position-specific analysis of DNA methylation within the EgDEF1 gene by bisulfite sequencing. A-C: PCR fragments F1, F2, and F3. The methylation percentage at every cytosine position within the sequence is represented by black bars. The symbol below the horizontal axis corresponds to the sequence context of the corresponding C: CG is a red disc, CHG is a blue square, CHH is a green triangle. N, M: inflorescence sampled on a clonal oil palm of either normal or mantled floral phenotype, respectively. The localization of PCR fragments on their respective target sequence is as indicated in Figure 1. n is the number of individually cloned amplicons included in the study for each experimental condition. (PDF) [file pone.0091896.s002.pdf]

Figure S3

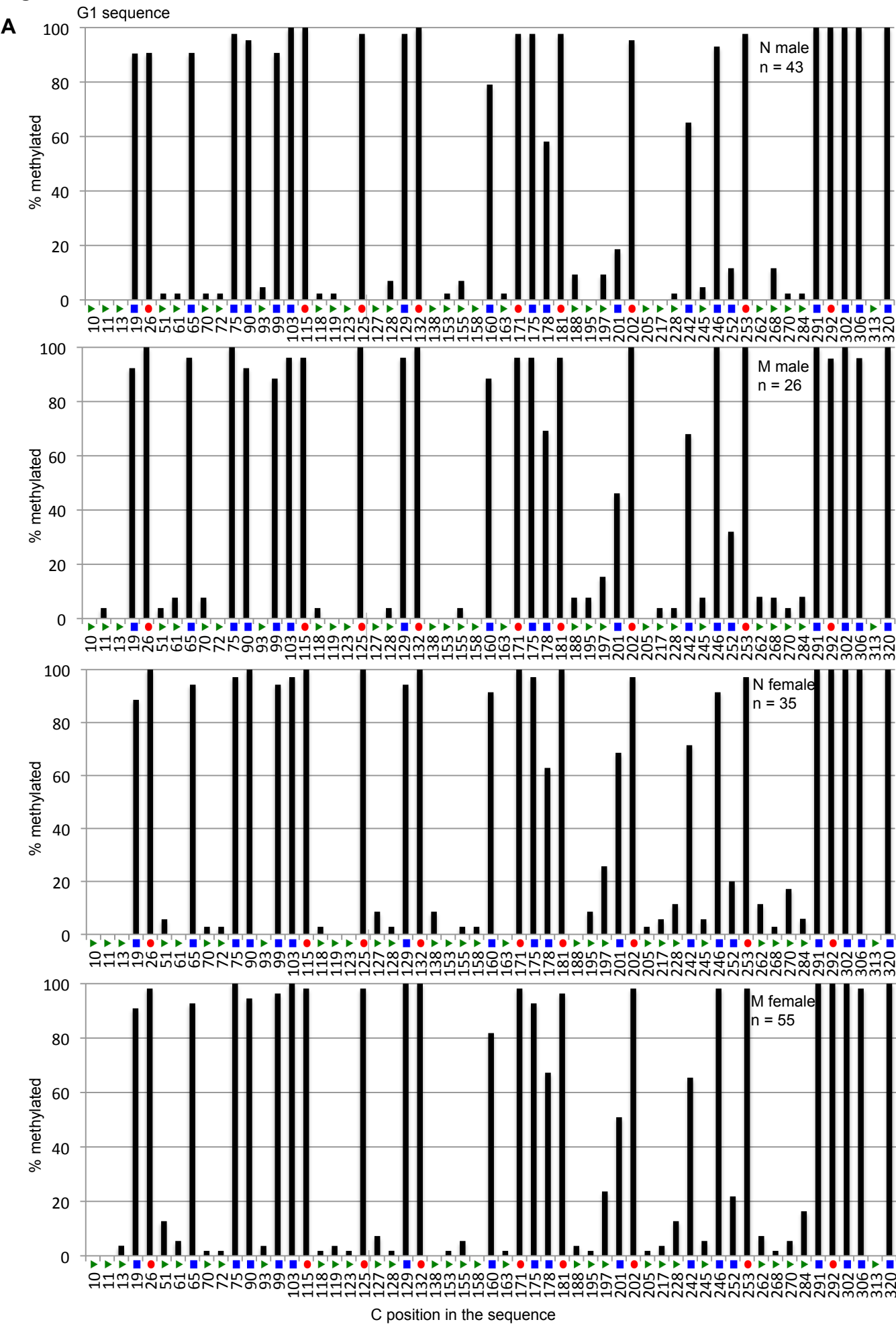

Figure S3

B

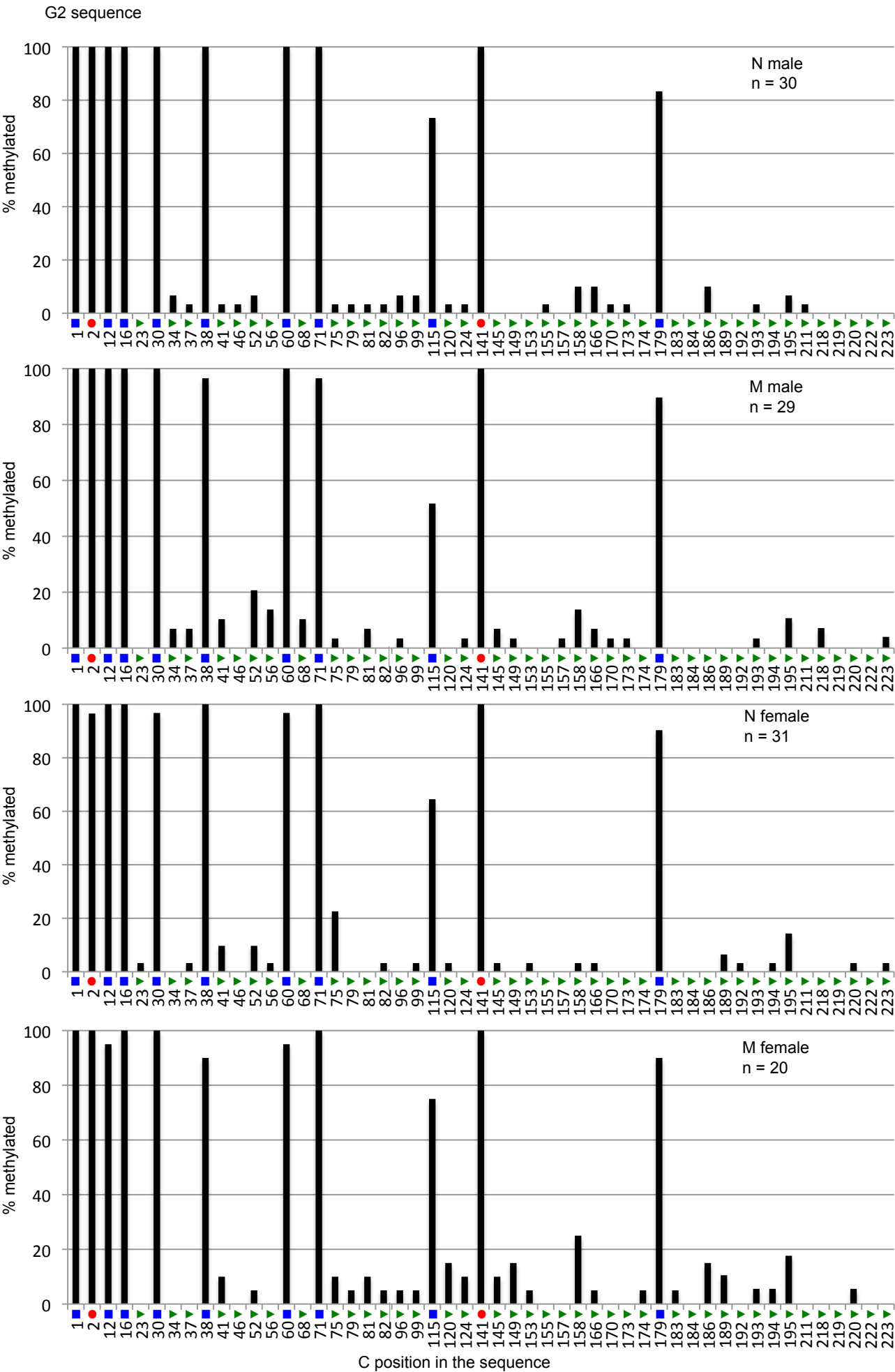

Figure S3

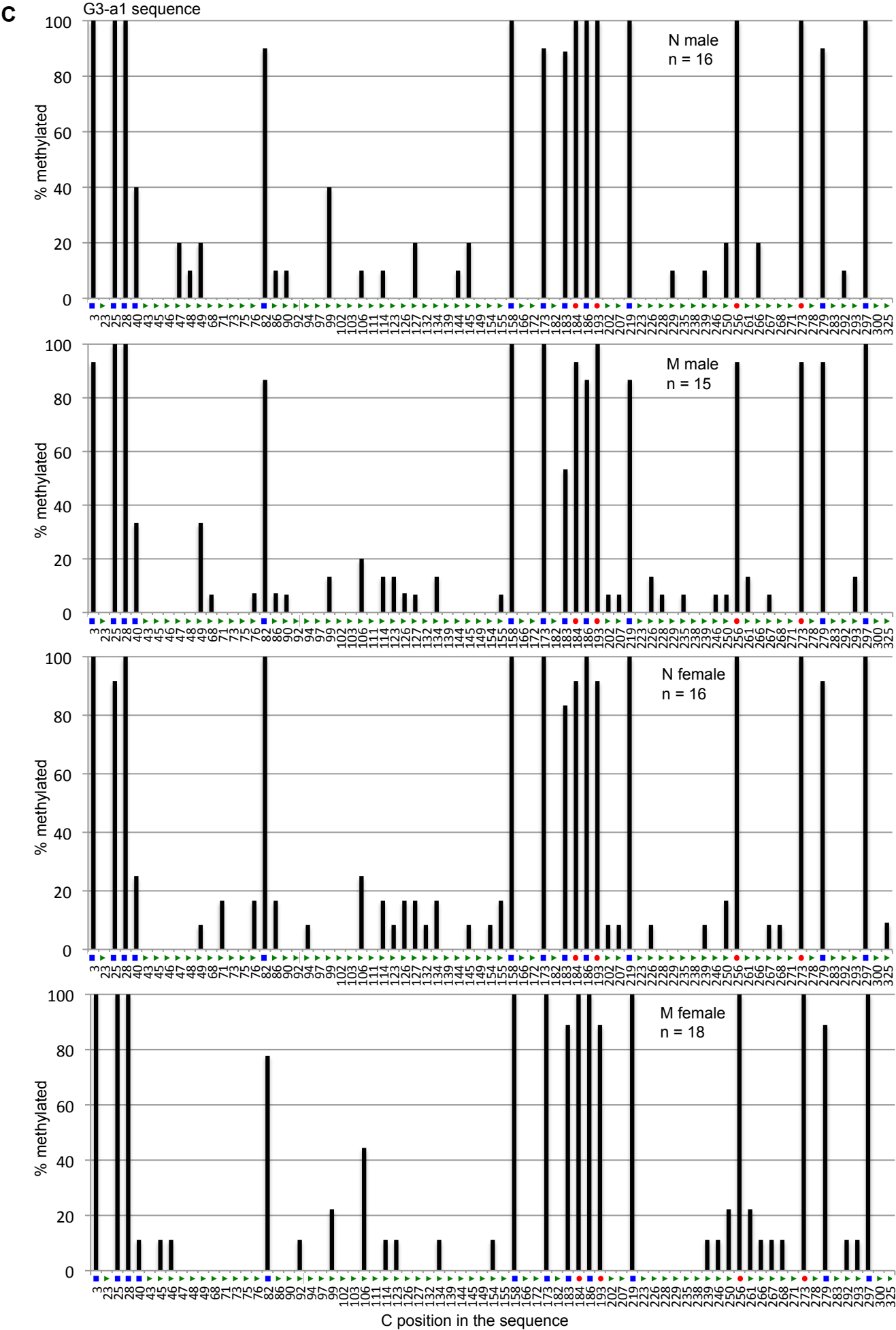

Figure S3

D

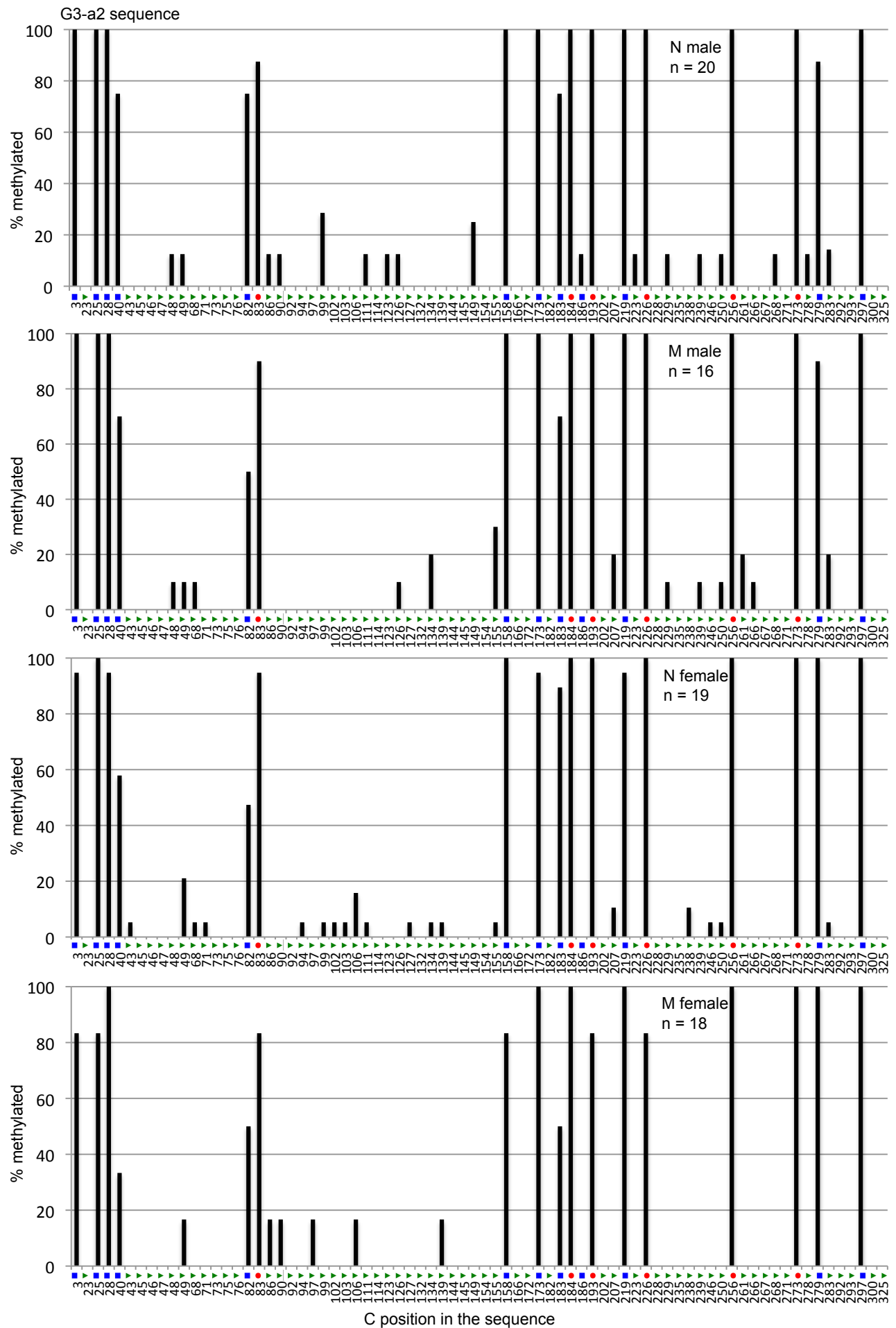

Supplement: Figure S3 — Position-specific analysis of DNA methylation within the Koala retrotransposon by bisulfite sequencing. A–D: PCR fragments G1, G2 and G3 (allele 1 and 2). For legend see Figure S2. (PDF) [file pone.0091896.s003.pdf]

Figure S4

A

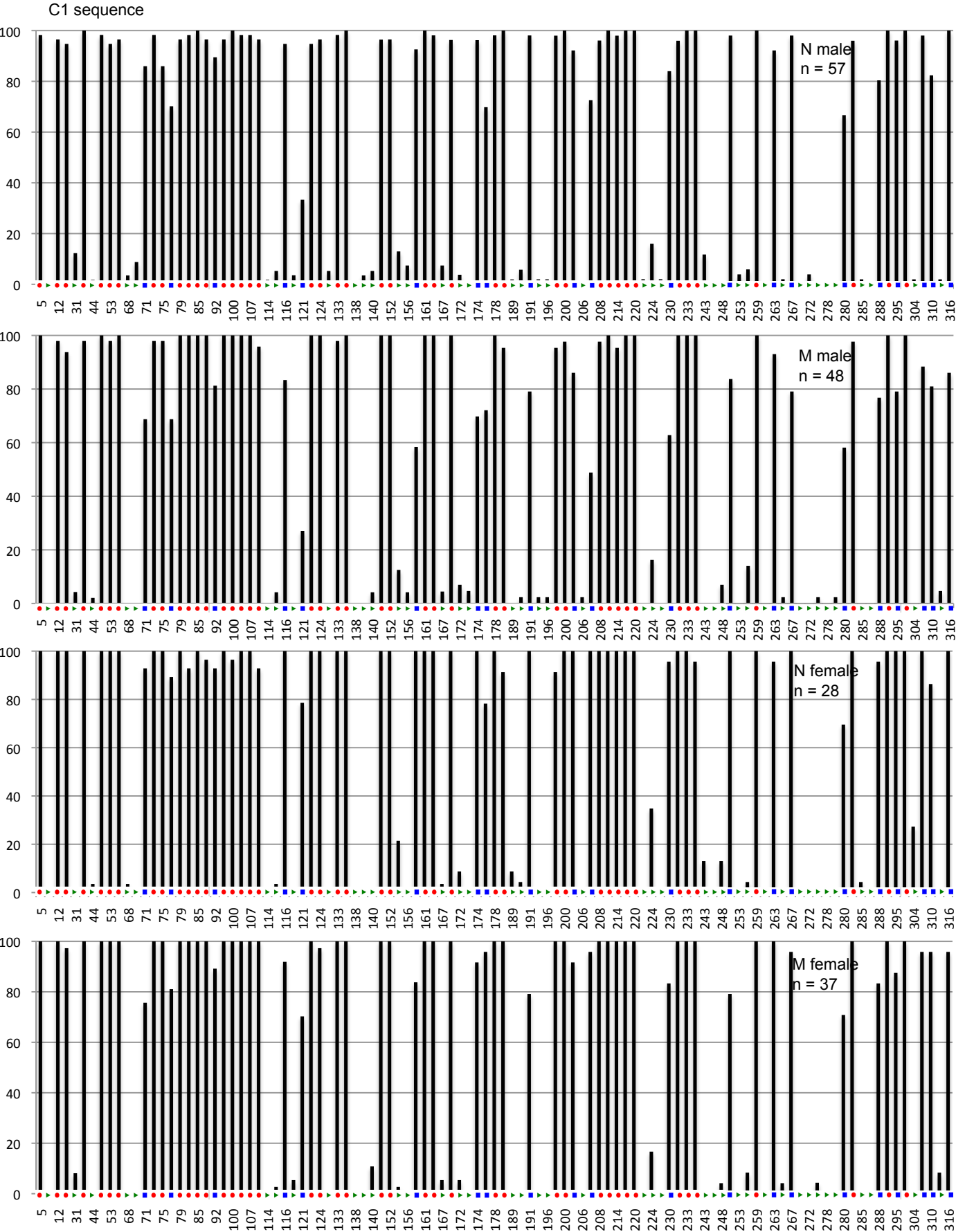

Figure S4

B

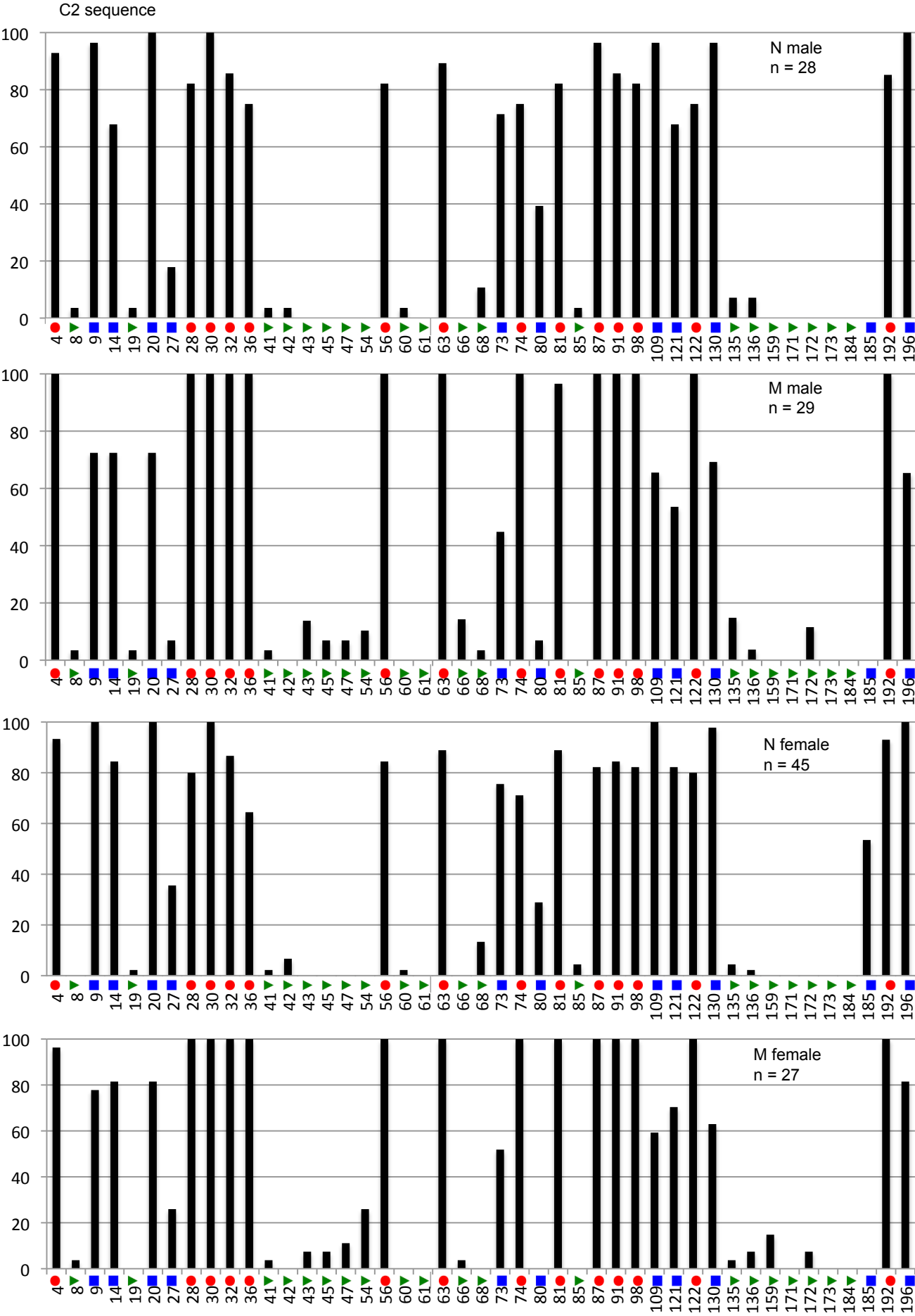

Figure S4

C

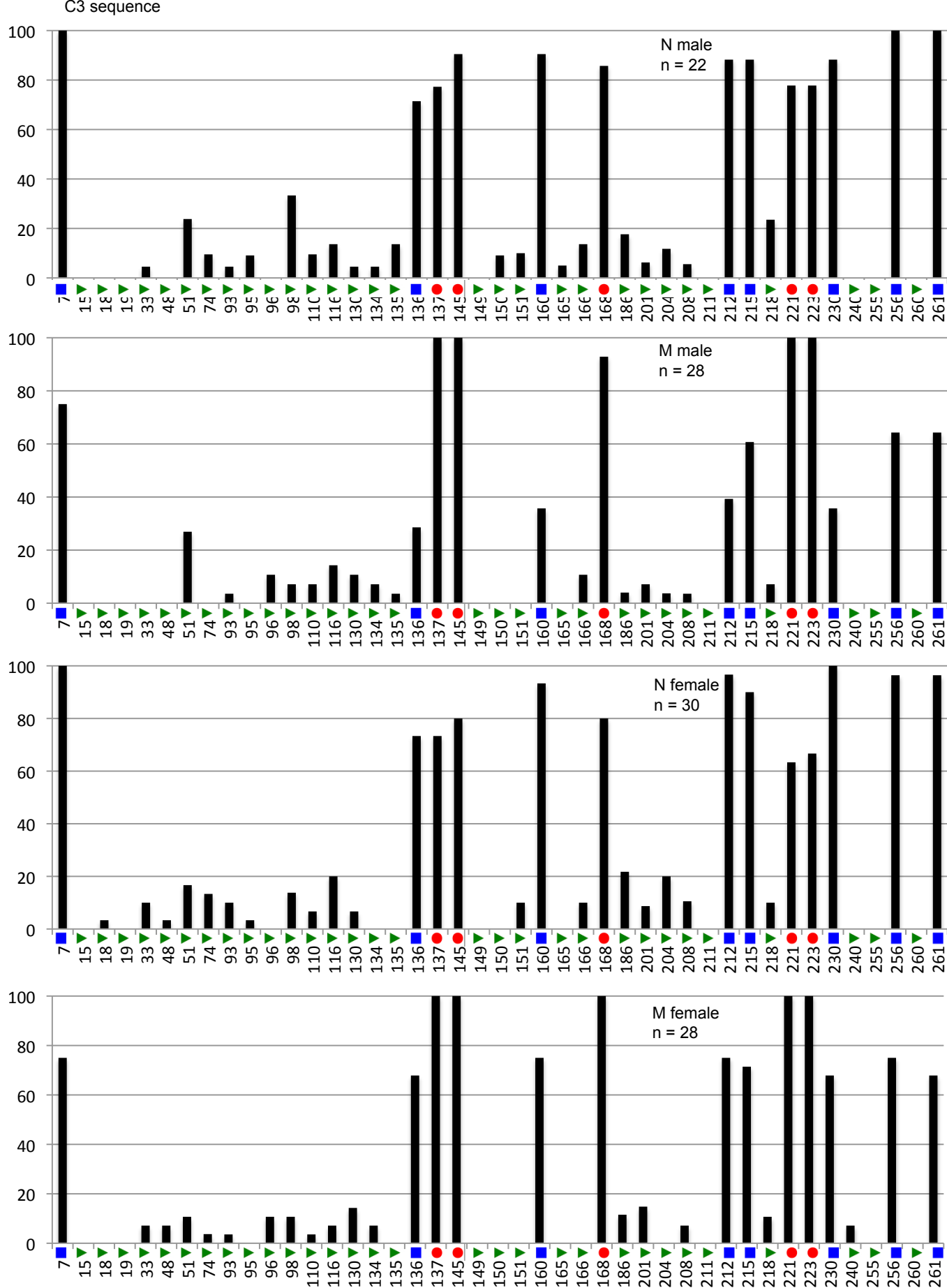

Supplement: Figure S4 — Position-specific analysis of DNA methylation within the Rider retrotransposon by bisulfite sequencing. A–C: PCR fragments C1, C2 and C3. For legend see Figure S2. (PDF) [file pone.0091896.s004.pdf]

**Figure S5**

**A Male inflorescences**

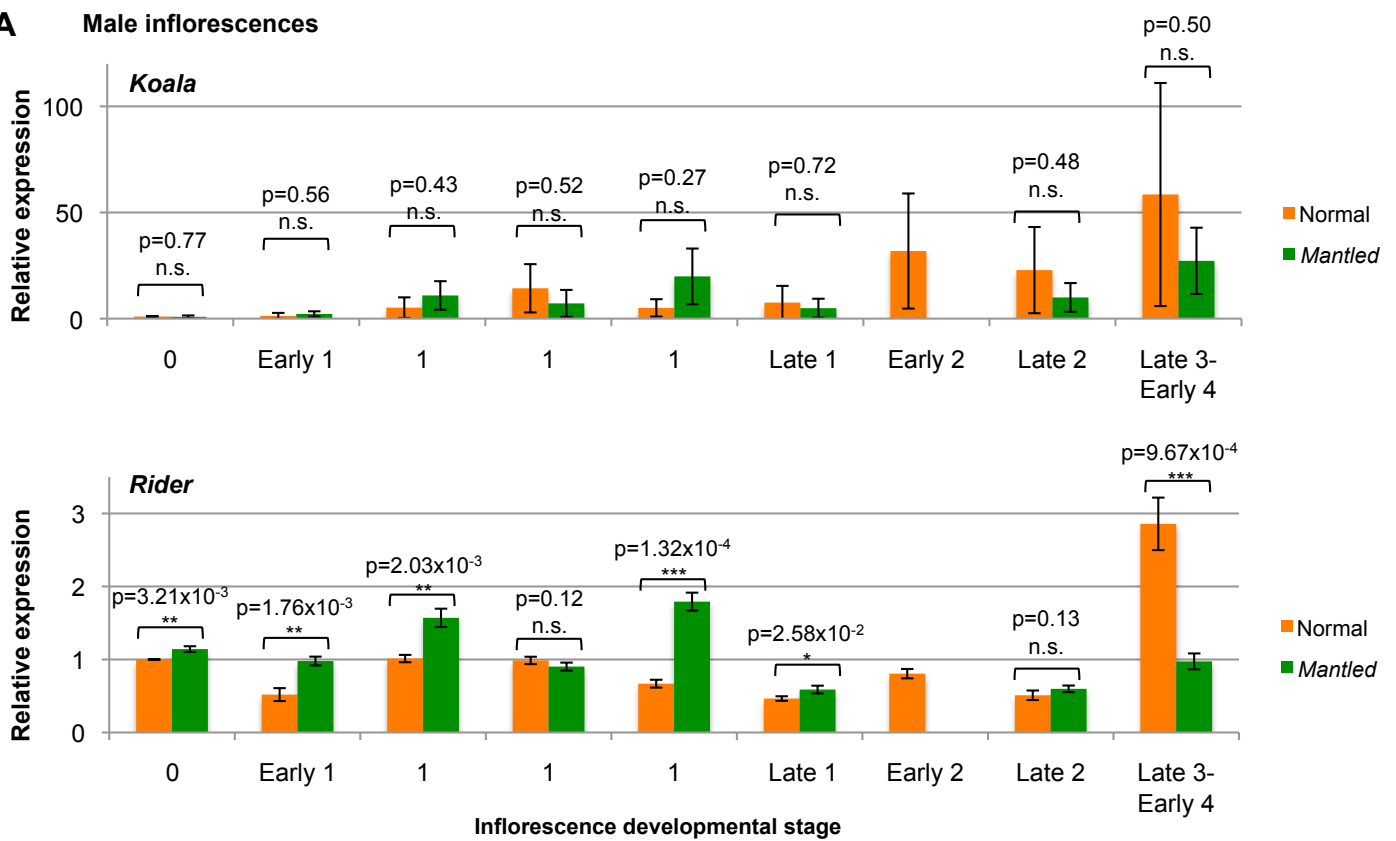

**B Female inflorescences**

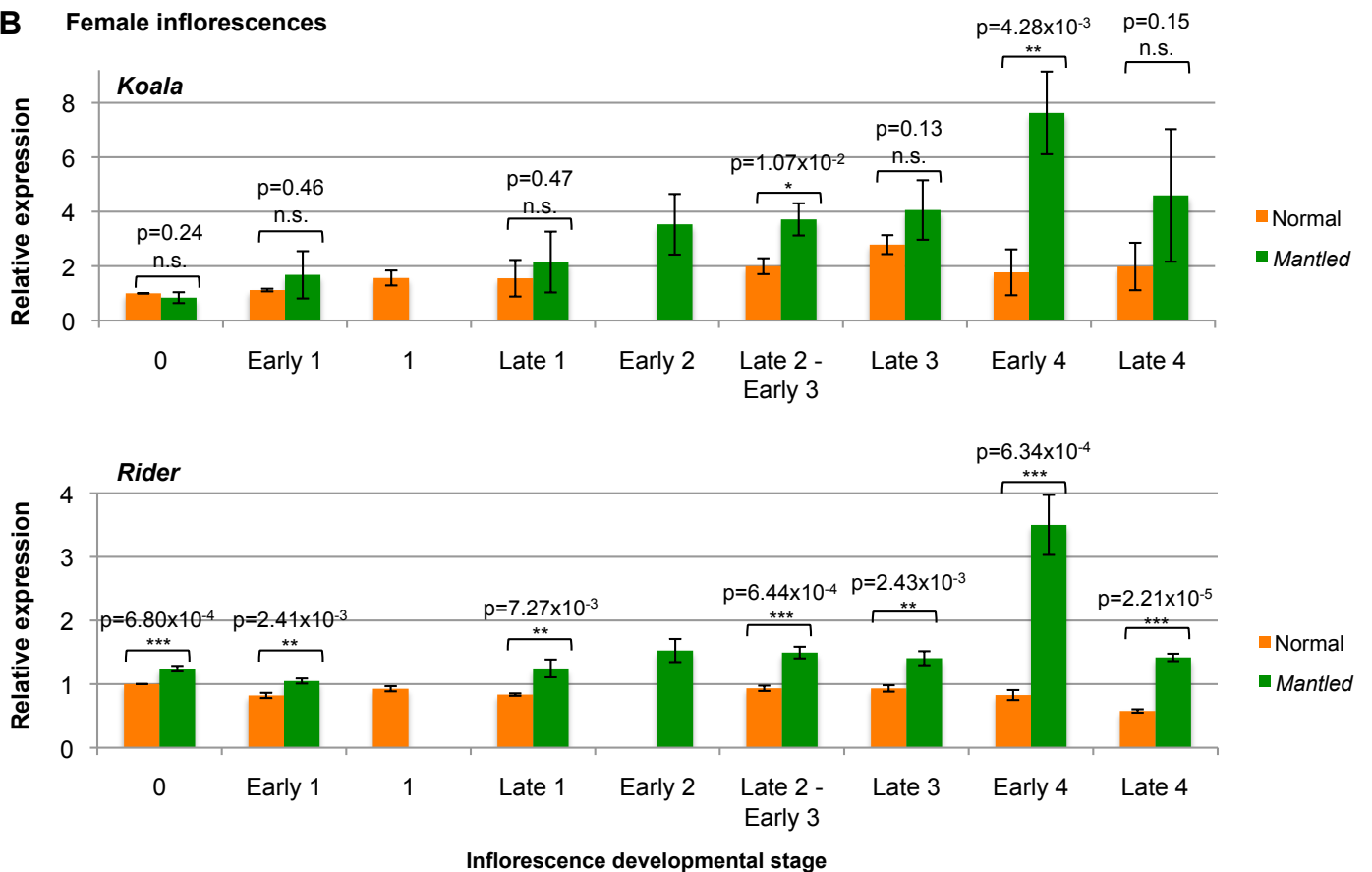

Supplement: Figure S5 — Real-time qPCR quantitation of transcripts produced respectively by the Koala and Rider retroelements. In male (A) and female (B) inflorescence developmental series of normal (orange) or mantled (green) phenotype, we evaluated the expression of the Koala (top) and Rider (bottom) retroelements. For each TE, p-values obtained through the comparison of the REs between normal and mantled inflorescences with Student's two-tailed t-test are indicated (n.s.: not significant; *: p<0.05; **: p<0.01; ***: p<0.001). (PDF) [file pone.0091896.s005.pdf]

Figure S8 Female inflorescences

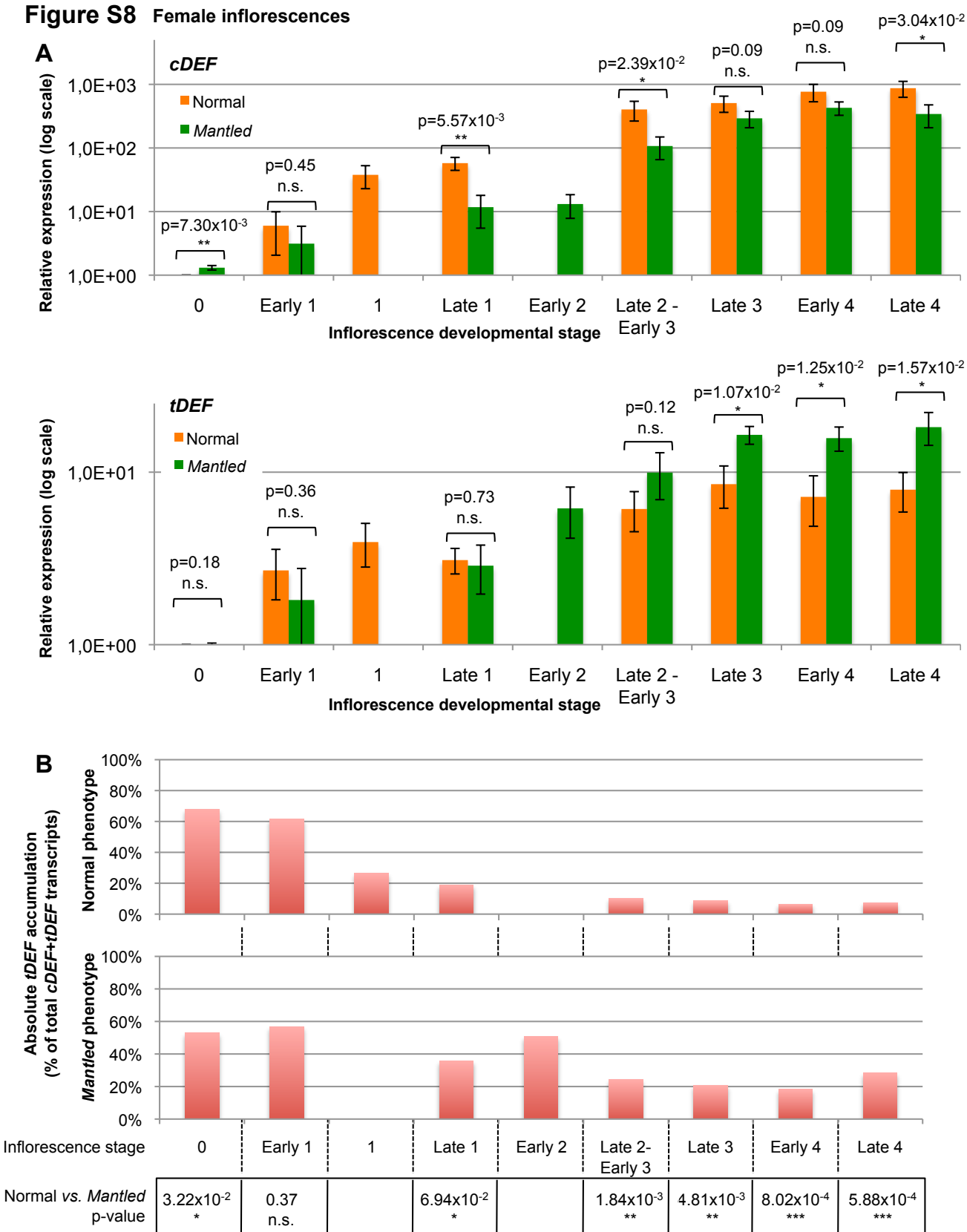

Supplement: Figure S8 — Real-time qPCR quantitation of cDEF and tDEF transcript accumulation in developing female inflorescences. See Figure 6 for legend. (PDF) [file pone.0091896.s008.pdf]
