# Supplementary material for: DNA Methylation and Expression of the EgDEF1 Gene and Neighboring Retrotransposons in mantled Somaclonal Variants of Oil Palm
Source: PLoS One. 2014 Mar 17;9(3):e91896. doi: 10.1371/journal.pone.0091896 (PMC3956824; doi:10.1371/journal.pone.0091896)
Supplement: Figure S6 — Localization of the rt-qPCR primers targeting the retrotransposons under study. A: Koala; B: Rider. Target Site Duplications (TSD) appear in italics; LTRs are in bold. Sequences are displayed according to the 5′-3′orientation of their respective ORFs. The complete list of primers is available in Table S5. (PDF) [file pone.0091896.s006.pdf]

Figure S6

A

Koala retrotransposon

*TGCAC***TGTTGGTACATGTTATATGACCTCATTATCTGGATACGTGGTGATAGATTATTATTTAAATCATAAATATTCTTTCAGATCACATCAGA**
GGTTTATTGATCTGCTTTTTCAGTCTGATATTTGATCGGCCTAACTCGCCCAGCGTTGTTCTTAAAATATGATACTCATCTCAGCTTATGATCG
ATCTGCTGCGAATTACATTGATCTCTAACCGACATGATTGTTAACTAAAGGGATACATAATATTTAGTTCAGCCAGTTGCCGATTTAAGCTATG
TCACATTGGTAAAATATCAATGTACCGTAAGAATGCAGACAGTTATCTAATAGCAGTCATCCAGCTAAGCATTAGCTATCTTTCAGTTATACTA
CAGTCAATCACCATGTGGGTAAATACTTCATGATTTTTATATAGCTGGTCATTCTATTATAATAAATTAACGATCTAACAAACTCTCCTAATAG
TCTAACAACCTTAGCAGGCCTCTTCATCCCCTATATATGAAGGGGGCAAGGTACCCTCCACAAAATAAGTCAGGATTGATAGAGTGGAGACTCT
GCTGAATTATTTTCAGCTCCCCCATTGAAAGAAATAAGAGTCATCTCTCCATTTACCGACAATCTCTCTTCTCATCCTTCTATTCTTCTATTTT
TACAACCTATTCTCAAGACTTTACCTAACTTATCCATCAGAGGATCTTGAACCAGAAGGTACCCGCAGGTTTCGAGATTTTCTTTGCAAGATTT
GTTACAGACTTCACCAAATTCAACCAAGAAACATTCTAGTTCGATTCAAGACCCATCTCGTTGCCTGTCTTTTGAAGCCTTGCAGCAATAGATT
AGTGTTAGAGGAAGGGCACTTAAAATATTTTAGTAGAAATGGTGAACAAGAAGGCACCAAATCATCCTTTACCTACTTCATCTTCTCCTGAAGA
RT1-QF1
TGTTCAGGAGAATACCTAACCTTCAATTATTATTGATCCACAATAGTTTAGTCTCTTGGTTCAGCAGGTCCAAGCTCTTACTGCTGTAGTATAC
RT1-QF1
GATAGAACGGTAGCAAAAAGTAGCCCCCCAAGTAGTCCAATCACATCACACTTAGCAACCAACTCCTCGAGATCTTCATCATGATCATCTTCTT
RT1-QR1
GACCATACTGATCACTCTTGGGAAGTGAAATCTGCTCCACATAAGAAGCCATGTATGGAGGAGGGTCTTGGACAAAAGATTCAAAATCTTGAGA
AGAAAATGATGGAGATATAGATTGGAAGCCATTCTCAAGGTAGAAGAGATTTCAACTTACGATCTCTTTTTTCTCAAAAAATTCTTGGTAAATC
AGTTCCTCCTCGATTCAAGATACCTACAGTGGAAACATTCGATGGCTCCATCAATCCCTTAAATCATCTTGAAGGCTTCAGAGCCATTATAAGA
CTACAAGGGGTATCTAATGCCCTATTTTGCATCACTTTTCCAATCACTCTCAAAACATCTGCAAGAATCTAGTTCTCTGACCTCCAGACATGAT
CTATTTCATCTTTCACGCAGTTAGCAAATATTCATCACGTATTTCGATAATAAATGGATTCATTTGAAAAATACTAGCAATCTTTTTACTATCA
AATAGTAGGAAGGTGAATCTCTTCAAGAATATGTGGCATGCTTTAATGCCGCCATACTAAAAGTGAAGAACTTCAATGAGTCCGTTGCAATGGC
AGCTTTGAAGCAAGGACTCAAGAGTAATCGCCTGATCTTCTCTCTTAATAAGAACTACCCAAACAATTATGAACAAATATTGATCTGGGTTTGT
AAGTATGCTCAAGCTGAAAAGAAAGAGAGCATGCATACTTCGTTAGGTGAAGAAAAAAAGAGATGGAAAAAAGTGACCCTAGGAAGAAGATCAT
AAAAACCAACAGGATAACTCTGAACGACCTCAGAAGAATCTGAAGTTCAAAAGCCCACCTCGGTGATTCGACATCTATACCTCACTATCTACTC
CTCGAGCTCAGATACTGATAGAAATCGAGGATTTAGGATATATTCGCCGACCTAATCCTATGAAAGCCCCTCCAGCAAAGAGAGATAAGAGAAA
ATACTGTCATTTCTATCGAGACCATGGCCATGATACTGAGGAGCATCGATAATTAAAAGATAAAATTGAAGTATTGATCCACTAATGTTACTTT
AGTAAATATGTTCGAGATCGGGCAAGTCAAGCTACTGACCAATGATAGAAGCCCAACAATGAGAATGATCACAACAAGTCTACTGCTGCAGTCA
RT1-QF5
TCAATATGATCTCAGATCTCGACGGAACTGAGGAGGTGGCTGAGATTACTGAAAGCCTTCCAAAGCATCAGTGCATGGATCACATAATTTCATT
RT1-QR5
TTTCGATGATGATGTTAGAGTTCAAACTCCTCATAATGATGCTGTTGTCATCTTTATGGCAATAGCCAAATATGATATAAAAAGAATACTTATT
GATAATGAAAGCTCTGCTAATATGTTGTTCTATGATACATTCCAAAGAATGAATATGATAGATTAGCTAAATAGGTTGGTACCCCCGTAATTGG
ATTTTCTAGAAATCCTGTAGCTGTGGAAGGAGAGGTCACTTTGTCCGTCACTGCAGGGATAGAACCATGCCAGTCAACTGTAAGACTCACTTTT
TTTATTGTCAAAATACCATCAGCTTATAATGCTATTCTTGACCGACCTAGGCTCAATGCCCTTAAGGCTATAGTCTTCACTTATCATTTACTAG
TCCAATTCCAAACTAAGTATGGAGTTGGAGAAATATGAGGAAATCAGATACTTGCTAAGTAGTATTTTCTGATAGCTGTAAAAATTAAAAGATC
TGTGGAAGCTCTGCCAATTAAAATACTTGATCAAGAAAGTAAAGTTGATATCATCAAAAATCAAGGAGAACCAGTCGAGAAACTTATTACTGTT
CTTTTGTATGATGATCCAAAAAAAACTGTCCAAATTGGATCTCTATTGAAGCCTGATCTGACGGAGAAATTAATATCTTTTCTTCGGATGAATG
CCGATGTCTTTGTTTAGTCAGTCTCTAATATGCCTGAGATTTTAACTGATCTTATTGTACATAAGCTGAATGTTGATCCAAAGCATAAACCAAT
RT1-QF3
GCAACAGAAGAAAAGAAGTTTCACCCCGAAAAGATAAAAAATAATAGATGAGGAAGTCGACAAACTCTTGAAAGACAAATTCATCAGAGAAGCA
RT1-QR3
TAATATCCAAAGTGGATTGTAAATGTTGTCATGGTTAAAAAGGCTAATGAAAAATGAAGGATCTATGTTGATTATACCGATCTCAACAAAGCTT
GCCTAAAAGATAATTTTTCTCTCTCAAAAATTGATCAGCTTGTTGATGCTACTTCAAGACACAAATTACTAAGCTTTATGGATACTTTTTCTGG
TTATATCAGATCAAAATGGCACTTGAGGATGAAGAAAATATGACCTTCATCACTGAAAAAGATTTGCATTGTTATAAAATGATGTCTTTTGATC
TTAAAAATGTAGGTGCTACATACTAATACTTGATTAATAAGATTTTTAAGTAACAAATTGAAATTAATATCAAAGTTTATGTTGATGATATATT
AGTAAAAAATACTGAGTCAGATCAGTATATTGCCAATCTGGAAGAAGCCTTTGGAGACTACGAAAATATCAAATGAAGCTCAATCCCAACAAAT
ATGCTTTTGGAGTAACTTCaAAAAAAtTTTTAGATTTTTTTGTAACTCAAAGAGGAATTAAAGTCAATCTTAAGAAAATTCAAGCTCTATATGA
AATGAAGCATCCGAGCTCCATCAAAGAAGTACACCAACTTACTGGATGAGTAGCATTGCTTAGTTAATTTATTTTCAGATCAGCTGATAAATAT
CTTCCTTTCTTTAAAATTCTAAGGCAGATCAAAGACTTCAATTGATAAGATGAATGTCAAGCAGCCTTCGATGATCTCAAGAAATATCTCGACA
CAGCTCCATTACTATCAAAGCCGATCAAAGGTCAAAAATTATTCATGTATCTATCTATTTCAGCAAATGCTGTTAGTTCGATTTTAGCTTGAAA
TGATGTAGGGATACAAAGATCCATTTATTATACAAATAAATTATTTAGAGATGTTGAAACTTGATATTCTAAAATTGAGAAGATAATTTATACC
CTCATTGCATTAGCAAGATGACTTCGATCGTATTTTCAGGCTCATTCTATTATAATTCTGATCGATCAACCTTTAAAATTAATTTTACAGTAGC
TTGATACTTTAAGAAGGATATTCAAATAGGCTATTGAATTAAATGAATTTGATATCAATATGGGCCTCGATCTTCATTGAAAGTTTAAGTATTA
GCTGATTTTATTATTGAATGCTCTATTCCTAATGAAGAACCAATCTTAAAAGAATTGAAAAATATCAAAGATTCTTATTTGATACTGTACATTG
ATGGAGCCTCAAATTCGAAAGAAAGTAGAACTAGTTTAATTCTAACGAGTCTAGAAGCCATTGTTAGTGAGCGTGCTTTGAGATTTAATTTTAA
TACTCAAACAATGGAGTAGAATATGAAACGCTAATTGTAGGACTCGAAATGGCTAAGGAGCTTAATGTTAAAAAAATTAAATTTTTTATTGATT
CTCAATTTATCGTCAGTCAGGTACGAGATTAATTTGAAGCTAAAGATTTTATGATGTTTCAATATTTATATAATATGAAAGAATTATTAAAAAG
TTTTGAAAATTTGAAGATCATGCAAATACCAAGGACAAAAAATGTCTGAGCTGATGCCCTATCTCATTTAGCCACATCGGATTTTTTCAAACTA
AATCAAGGTGTTCTTATTGATATTCTTAAAAAGTCTAGTATCGAGGCCTTACTAATTGTCCAAATTTAATATGAGCTAAGTTGCATTGATCCAT
TAATGGAGTATATTACTAAAGAAACTCTACCGGTTGATCCAGTCGAAGGAACAAGAATTAAAAGACAGGCCCCATGGTATGTTGTTCAAGATAA
TCAATTATATCAGAGGTTATATTCACTCCCACTGCTCCTATGCCTAAGGCCATCGGAAGCTGATTACATCCTTCGAGAAGTTCATAAAGGAATC
TCTAGTAACCATTTGGGGGGCAAAATTTTAGCTTATGAAATAATTCAGTAAGATTATTATTGGCCTACTATTCAAAAAGATTCAACCGACTTTA
TTAATAAGTACTGCGATCAGTGTCAAAGGTACGTCAATATATAACATCAGCCTACAACTGAACTTATTTCTATTACAGTGCTGTGGCCTTTTGC
TATATGGAGAATGGATATTCTGGGACCCTTTCTAATGACCATGGAGCAAAGAAAATTTTTAGTTGTGACTATTGATTATTTTACCCAATGGATA
GAAATTGAATCTCTGACATAAATCACTGAGAAAAAAATATGAGATTTTATGTGAAAAATAATTATTTACCATTTTGGTTTGCCTTGAGTTATAAT
TACTGATAATGAGCAATAATTTGATAATCAAAAATTTAAAAAATTTTATTCTGAGCTCTACATAGATCATATATTTACTTCGATTGGACATCTA
CAATCAAATGGAGAAGCTAAATTTATTTATAGAACTATCCTTCAAGGCCTAAAAATAAAACTCATTGAGGCTAAAAGTCTCTGGGCAGAATAAT
TTTATAATATTTTATGGGTATACAGAACTACACCTCAGAGACCAACTAGAGAAATATCTTTTAAACTTACTTTCGGAACAGAGGCTATAATACT

AGTCGAGATCGGTTTACCTACAACCGGAATAGAAAATTTTCATGAAAAAAATAATTCAGATCAACTGAGGACTGATCTCAATTTATTTGAGAAA
ACTCGAGAAAAAGCCCAGATTCATATGGTAGTTTATAAACAAAAAGTAGCCAGATATTACAACTCTCAGGTAAAGCCTAAAATTTTTTATAAGG
AGATTTGGTTCTCCAATGAGCTGAAGTCTCAAAGCCAATGGAGTAAGAAAAACTCATCCCGAATTGGGAAGGACCTTATCAGATTACTGAGGTG
ATCCATCCAAGAGCTTATCGAATTAAAAATTCAGATGGTACGATCATTTCGAGAACATGAAATATTAAAAATCTCAAAATGTATTATCAATAAA
ATCTTTCAATAAAGCATTCGATTTCTATCCCAATGAAGATAAAGTTCTGATCAAAAGTTCCATTGAAGAATTCTTTTTCATCCAAAGTAGATTA
GTAAAAATCGATCAAATGATGATAATAAGATTTAGGTGTCCAAATGAGATCAGAAAAAGTCAAACTAAGGCTACAAAAATTCCAACCACATTAG
GATGAGATGATGTGAGACATCCAATCTTCGAATTGGATCTCACAAGATGATATAAGACATCCAATCTCTGGATTGGACCTCATGAGATGATAGT
GAGATATTCGATCTTTGGATTGGACCTCACGAGATGATAGTGAGACATCCAATCTCCAGATTGGACCTAACAATATGACATTGGATACTCAATC
TTCGAATTGGACCTTAAATAAAAAACCTAAAATACTTGATCTCTAGATCAGGTTTCACAGATAATAGGATAGACTCAATCTGACAAGAAAGCAC
TCAGATCTATAAACTGGATTTTGCTATATCGATTTAAGGTGATTAATAATGAAGATCACAAAAAGACAGCATGGGTCATAAATCCACGAATAAA
AGAACCCGACTTGTCACTTCGAACTTAGATAAAGGTACTTTTAGAACCTTTACAGTGAATTATGAGAGATTAAGTTATCTATAGTTAGTTGATC
TGATTCAATTTCATTCCATAACAATCATACTTGTTAAAGTTAATCACATTGACTTCGTTATCTACTTGGTTTGAGAATAGTTAATATTTGATTA
CTTACCTAACCAAGGTTGAATTACATATACTCATAATCATCTAACCGAAGTCAGAAAATAACTATCTATCGTGAATGATATTTTTTGAGACATC
CAACCCAAATCGGATCATCCAAGATTAGTATTAGTTAAAAAGAAAAAATATTGACATTGAAAAACTCATTCATTAATGTAAAAAGTATTTATAG
AAGCCCACATCGGGCTAATACAAATGTTTAGAAAAAAAGAAGAAGATAATTCAGGCCTTAGCTTGGGAGTTATTTGTTGGAGGAGTTGAGGTAA
GGTCTTTGCCGACTGATTTCTTCGGAATAGCTTCGATGATGGAGGCCTCGATCGGACTGTTTGGGACTTCGATCGGAGAAACTCCAATGATAGA
AGTGGAAGCAAGCTCATTAGTTGTTGCAGAAGTCACGTCGGCTACTACTTCTAGAGCTGCTTCCATTATGACCTTGCTGAGATCAAGGTTAGGA
AAGAGCTTGATAATTAAACTCCTGTAGCTCAAAAAGCCATAATTGAAAGCTCCTTTTGAGGCCTCAGTAACCTCATCTTGAAAAGTGTCGGAAT
TCTTATAATCTTCAATAATCCTTGACATCCACTCTTTGTCTTTATCAAGTTGTCTCTAATATGGGACAAGAGCTTCATCATGCTTTTCAATTAT
TGACTCGACCTCCTTCAGCTTGGCCTCCAACTTTGAGATTTTTTTCAGGCTGTTTTTACAACTTGTCATCTTTATCATTCATCTGTCATTGGTT
GTCCAGTCTCCTTAATGGAATCTATTTTTTCTTGGAGCTGTTTCTTCAGCCCCTTCATCATTTTGCTTTCTTTGACAGCCTTCTTCTTTACTGC
CTTCTTGACCTCCTCAACTGCTTTTTTGGCCTCTTTCTTGGCCTTGAGCCTCTCTTTCaAAAAAAAATTTATTTTTGAAGAACCATGTCAAGTG
CTCATTGAGCAACTTGAAATACACAACCGACTGCAAAAAGAAAGATATATTCAGATCAACAAATAGCCAATGTAAAAAAAAAAAAGAAAAAGAA
AGAAGAGAGTGGAAGAGAATGAAGACTCACGTTCATGATACTGTTGAATCCATAATTTATTATTTGCTCCAAAGAAAGATCCCTCCTATCAGCA
TCTAGGTCCTTTTGAGGCATCATTGTACAGAGCAAGTCCTTGCCTAGTTTGGGTGTCTCCAAAAGTGATACCTTCGCCAAGTCAATTTTTTTTG
ATGAGAAAGTTGGTGGGTTGACATCAGGAGTCTATGACCTCGGATGCTGATTGCTCAGCCAAACTAACTTGGCCGATGTTGGAGGTGTTGGGGA
AGATGCCTACTTTGGACGTGCAGACTCTTTAGGAGGTGGACAAGCCACCTTAGTAGGTTGATGAGTTGATTTTACTAGTGGTATAGTGGAGATC
GATGCAGCATCTATGTCAATTAATATTGGCCTAGGTGATGATCGGGGGCCAATCGATCTTAGAGGAGCAGTCTCCTTACTCTTCTTGAGAGCAA
TCGGTTAGATCCTTCTTCCTCTTCTTTGAGTTAGCCAGCCTTTATAGGTTGAACTTCATTATTGCTACTATATAAGGAAAAAGATTGTTAGAAA
AGAAAATTGATGAAAAATGATAAAGCAATAAAGAAAAAGAATTAACATCAGACTTATCTTAAGGAGAGATCTGACTGATGTCGGTGTCAAACAA
GGACTGATTGGACACCAATAGGCTCAACTTGGATACTTTCATATCTAAAAGCTCTACAAAAGTCTCCTCATCCTCAGATAATATTTTGTTATTT
GAGTTCCATTTGGTTTTAGGCTTAGACTATTTACAAATAAAACCCTACGAAACATTAGAAGAAATAAAGAAAAAATAATTTTTTCAATTATGTA
TTGAAAAAGGAAGAGTGGGACCGAATTTATATTGAGGCCGAGGTAAGAAATACCATCAATTTTTTTCATAAGGATATTTTCTTAGGATGACCAG
CATCCTAAAGAGAGAAATTCTTAGTTCTATAGCAGAAAATTTATGATAGATAATGAAAACTATGATCATCCTAATGGCATTGGGAGTAACCTAG
ATTGGATGGAGTTTATAGAAGTCTAAAACATTACTGAAGAAAGGATGTAAGAGAAATTAAAGTCCAGATCAAAGTGCCTCATCATAAAATGCGA
CATAACCTTCTGGAGGTTCAATAATCCTACCATCCCAACTTGGAGTTATAAGTTGAATCGAATATTTTTTCTTAAGTTCCTAAAGATCGAGATT
GGTCAGGACAGATTGTTTAGTACTTGGGCCGAAATAATCTAAAAAATCTCTTATAGGAGATTCTAGATCATCCTAACCTGAGGTACTAACTTCA
GCTGTTGTCCGAGATAGATTTTCCATCTCAAACTTGAGCAGTGGTTGGGTTGACCAAGATATCCATTAGTCCTGACTTATCTCATTAGTCCTAT
CAAAAGATATGTTTGAAAGGAAAGAAGAAAGTAAAGGTGAATGGGAAAAGAGAAACAGaAAAAAAAAGaGaGAAAAGGGAATAACTATcCaaaa
aaaaaaaATAAAGATTGTAAAACCTAAAATCTCAGATGTTCATGGAAAAGAAGTTGAAGATGAAAGAGTCTGCCTAATATGTAAAGAGGAATTT
GAGGAAGCTTTTAAAAGACTTCTTTACTTCTTTCTTCTCCGGCTCCAATGACTTTGGGGGGTCTCTACTCCGATACCAATGATGTTAGGGGCTC
TGAAAAAATTTCAGCAAGATTGTCATGTGaAAAAAATAGTAAAGTAATTTTTGAACAGAGTATAGATCTATTTATAAAATTTACTAATAGTCTC
GATCCATTTGATATGCCATTAGATCAATGTCACTTGTCAGTTATCCGATGGTTCTGCAGAGAGTTGTTGCGACGCACCTCTTCTTGATTAAGCC
ATGCAAGCAGGATCCATGTGATAGCTGAAGTGGCCAATTACAGTATGACACCTAGCACATACACATTAGGGACTAGCCACCCAAATAATTGTGA
GCTCTCGATTTTCTAAGCGCTCATCATTACTAAGTTCAACAAATCATTTTCAAGAAATTGTGTATCACTTTGAGTGATTAAAAAATTAATACTT
CCAACCATCGTGGTATGGAGCATTTATGATGACAATATAAGTCAGATGAGATCTTTTGAAGAGACTTTCCACACCCGATGTGAAGAACTTCACT
TCGGTGTGAAAAATGGAGGGCATA**TATTGGTACATGTTATATGATCTCATTATCTGGATACGTGATGATAGATTATTATCTAAATCATAAATAT**
TCTTTCAGGTTTCATCGAATGTTTACTGATCTACTTTTTCAGTCTGACATTTGATCGATCTAACTTGCTAGTATTGTTCTTAAAATATGATACT
CATCTCAGCTTGTGATCGATCTACTATGAATTACATCGATCTCTAACCAATATGATTGTTAACTAAAAGGATCATAATATTCAGTTCAGTTAGT
TGCTGATTTAAGCTAGTCACGTCGATAAAATATCGATGTACTATAAGAATGCAGACGGTTATCTGACAGCTATCATCTAATTAAGCATTAGATA
TTTTCTAGCTATCCTATAGCTAATCATCACATGGATAAATATCCTATGATCTTTATATAGCTGACCATTCTATTATAATAAATTAATGGCCTAA
TAACTTGAGCAAGCCTCCTCACCCCTCTATATAAAGGAGGGTAACATACCCTCCATAAGGTAAGTCGGGATAGATAGAGTGGAGACTTTGTTGA
ATCATTTTCAACTCCTCCACTGAAGGAAACAAGAGTCCTCTCTCCATTTGTTCACAATCTCTTTTCTCATCCTTCTATTCTTCCATTTCCACAA
TCTGTTCTCAAGGCTTTATTTGACTTATCCATTCGAAGGGTCTTGGATCGGAAGGTGCCCCACCGATTTCGGGACTTCTTTTATAGGATTTGTT
GCAGACTTCTCCAAATTCAACTAAGAAACATTCCAATTTGATCCAAGACCAATCTCATCGTCTGTCTTTTGAAGCCTTACAACAACA*TGCAC*

B

Rider retrotransposon

*GATAT***TGTTAGAATTTGATGCCTCAAGATTCAGCCCACATTGAGTCCACAGTGAGGTTCGCGACGAAAAATGGAGTCCAACGAGATCAAGATCA**
CCCAAAACGGAGCTCGGATGGAGGAGATACGAGCTTTTGAAGTCGGCACGAGATTCGAGGCGGTGGAGGACCACCGGCGACCGGCGGCGGGCGG
CAGCGGTGCGCGGGATGCGTGACCCAGGCCCGCGTGGGACACGCGACCCATGTGGGCGGGCGGCCCAGGCGGGCGCACGGCCCAGCCGGGTGGG
CGGCCCAGTCCACGCGCAGGCCCGTGCGCGGGCGCGGCCTAGGCCCGCGTGGGCGGGCAAGCCCAGGCCAAGGCGGCCTGCCTGGCCCTTTACC
CCGGTCCACCATGGACTGGGCGGTCCAGGGCTGGGCCTGTGGACCACGCGAGCATTTCCCACACATTTTGCACGGTCCACGGCACTATTCCGTG
GGCCGATCACGATCGGAGGGCGTGGGTTGATCTGTTTTTTGATCCGATGGTTCAGAACCTAATTTGGGTTGATTAAGGATTCTTAGGTTTTAAC
CCTTTAAAAGGGCCTGTGGACGAACAGTAAAGTAGAGGTTCGGATTTTCTCACCGTACAGAGCCGTACAAACCCGATTGAAGAGAAAGAGGCGA
AAGCCCTGAGAGAGAAGGAGCAGGAGGCTCCTGGACAGCGGTCGTCAGGCACTTCAGGGGTTCAGGGGGTCTTCCAAGAGAGAGAGAGCTTTTG
TGAGGGAAACTTCTAGTGAGAGAGAATTGGGTGTACAAGGGTTGAGGATGAGGTCTCCTCTTGTAAAATTTCTTTTTCATAGTGAAGTTTGCAT
GCCCCATGGAGGCGAACCCTTTTGTGGCTGATCCATGTATTTTGATTATTTTTCTTTTTGTTTTGTATCTTCTTTCTTCCTGCTGCATCGCATG
GTACTGAAAAGATCTTGGGAGGTGGTGTCCTGGCCAGACATCCACCCAACAAGTGGTATCAGAGCAAGGCGATACAAGGACGTAGATTGCAGTG
GTGGTGAGCAAGACTGAAGATGGAGAAGACAGGAACAATCAAGATGGAGATCAACAAGTTCGATGGTAAGAGCAATTTCTCCTTGTGGCAGGCA
RT2-QF1
AGGGTGAAGGACGTGCTCATCCAACAGGAGTTGATCGATGCTCTCTTGTGTGATGAGAAGCCGACCACCATGGAGGTGCGAGATTGGAAACGGC
TACAGATGCAGGCGGTGAGTACCATCTGTATGTACCTGGCGGATGAGGTGGTGATCCATGTGCTGAGCGAGACTTCTCCGACGGTGCTGTGGTC
RT2-QR1
GAAGCTCGAGGAGTTGTACATGGCGAAGTCTCTCACCAATACACTTTTTCTCTGGAGGCAGTTCTACCAACTGCGGATGATTGAGGGACAGAGC
ATGCAGGAGCATCTGAGCCACTTCCAGAAGATCCTCACCGACCTCCTCAGCATTGGCAAAAATGTTGAGGAGAAGACCAGGGCACTGGTTTTGC
TGGCGTCGCTTCCCCCTTCGTACGAGTCCGTAGTAACTGCTCTTCTAGTAGGGAAGAGCACTATCAAGATGGACGAGGTCACCACGGCGATACT
CCAGAATGAGGTTCTCAGAAGGGAGAACCCAGCTTCGAGCTCAGGTGGTGGTAGCTCAGCTTTGGTGGCTTCTGGAGGAGCAGGATGTGGTAGA
CGGAGCGACAGGAGATCGCAATGAGGGCGGTCTAAGTCCAGGAGGAACTTGAGCAAAATCAGGTGTTACCGGTGTGAGGAGTTGGGGCATCTAG
CCAGAGATTGCCCTCAACTTAAAAATCAAACGGTGGCTGCTGTAGCGACGGCCGGCAGCGATTCAGATGGAGATGTCCTGGAGATATCTGATGA
GGTATCTACTTCTTTCCAGCAGTGGATATTAGATTCTGCATGCCCCTATCATGTATGTTGTAGAGAGGAGCAACTTGACTCCCTGGAGAACAG
RT2-QF3
GAGGGCATTGTATATCTGCCGGATGGATCGAGCTGTGCGATCAGAGGCATTGGGACGATCAGCTAGAGGACACATGACGATGTAGTGAGAAGAT
RT2-QR3
TGGGAGAGGTCCGATACATATCCGATTTCGGGCAGAATCTTATCTCACTTAGCAGACTGGATTCGAGAGGCTACAGGACGGTAGCTGGCGGAGG
AATCCTGAGGGTGTTACGTGGCGATAGGATTGTGCTGGAGGGGAAGAAGGAGAGCAGATGACATTATTACCTGACAGGAAGCCCAGTGCAAGGT
GGAGCTTTGGGAGCCAGGTGGAGTCCAGAGCGAGGTGGAGCTCTAGGAGGCGGATCGGGCACGAGACAGGAGACTCGGGAGGACGAGAGGCGAC
GTCGCAAGATGAGATTCCTATTACCGCAGGATGATGCCCCGAGTAGGTCTCAGGTCAGGAGGAGCACAGCATACGACAGAGATGGGATCAAGCA
GCCTAGCTCGACTCTCATGTTTGCCCATCCATGATCAGCAGGCGATTGCCCTAGGGCATGGGGGCGAGGAGATCCAGAAGCTCTCAGAGTTTGA
AAGAGGCCGAATATCGAGTCGTGGTGGAGAT**TGTTAGGATTTGATACCTCGAGATTCAGCCCATATTAAGCCTACAGCAAGGTTCGCGGTGAAA**
AATGGAGTCCAACGAGATCAAGATCACTCAAAATGGAGCTCGGATGGAGGAGATACGAGCTTTTGAAGTCGGCACGAGATTCGAGGTGATGGAA
GACCACCGGCGACCAATGGCGGGCGGCAGCGGCACGCGGGACGCATGACCCAGGCGGGCGCGCGGCCCAGCCGGCGACCCACGCGGGCAGGCAG
CCCAAGCGGGCGCGCGGCCCAGCCGGGCGCGCAGCCCAGTCCGCGCGTAGGCCCGCGCGCGAGCGCGGCCCAGGCCCGCATGGGCGGGCAGGCC
CAGGCCTAGGCGGCCTACCTGGCCCTTTACCCCGATCCACCATGGACCGGGCGATCCACGGCTGGGTCTGTGGACCGCGCGAGTGTTTCTCACT
CGTTTCGCACGGTCCACGGCACTATTCCATGGACCGATCGTGATCGGAGGGCATGGGTTGATCTATTTTTTGATCCGACGGTTCAGAACCTGAT
TTGGGTTGATTAATGACTCTTAAACCTAATCTAATTAGGTTTTAACCCTTTAAAAGGGCCTGTGGACGAACAGTAAAGTAAAAGTTCGGGTTTT
CTCACCGTACAGAGCCGTACGAACCCGATTGAAGAGAAAGAGGTGAAAGCGCTGAGAGAGAAGGAGCAGGAGGCTCCTGGATAGCGATCACCAG
GCACTTCAGGGGTTCAGGGGGTCTTCCAAGAGAGAGAGAGCTTTTGTGAGGGAAACTTCTAGTGAAAGAGAATTGGGTGTACAAGGGTTGAGGG
TGAGGTCTCCTCTTGTAAAATTTTTTTTTCATAGTGAAGTTTGCATACTCCGTGGAGGCGAGCCCTTTTGTGGCTGATCCACGTATTTTGATTG
TTTTTTTTTTATTTTGTTTCTTCTTTCTTCCTGCTGCATCACGTGGTACTGAAAGGATCTTGGGAGGTGGTGTCCTGACCAGACATCCACCCAA
CA*GATAT*
