# Supplementary material for: DNA Methylation and Expression of the EgDEF1 Gene and Neighboring Retrotransposons in mantled Somaclonal Variants of Oil Palm
Source: PLoS One. 2014 Mar 17;9(3):e91896. doi: 10.1371/journal.pone.0091896 (PMC3956824; doi:10.1371/journal.pone.0091896)
Supplement: Figure S7 — Alignment of two transcripts produced by the EgDEF1 gene. Genomic: genomic sequence (note that only the 5′ and 3′ extremities of intron 5 are represented on this figure; the missing part of the genomic sequence is replaced by a double slash symbol at each gap border). cDEF: full-length EgDEF1 transcript; tDEF: truncated EgDEF1 transcript (see manuscript for details). Start and Stop codons are in bold, 3′-UTR regions of each transcript are in italics. Exons are numbered from e1 to e7 and introns (shaded in grey) from i1 to i6. The sequences matching the rt-qPCR primers used to amplify each transcript (Table S5) are underlined. (PDF) [file pone.0091896.s007.pdf]

Figure S7

```
                                                 -24                     +1
Genomic   AAGAGACCTTCCTGGGTGCTTGAGAAATAGAGAAGAGAGAAAAGAGAGTTGGAGATGGGG
cDEF      ------------------------------AGAAGAGAGAAAAGAGAGTTGGAGATGGGG 30
tDEF      ------------------------------AGAAGAGAGAAAAGAGAGTTGGAGATGGGG 30
                                        -24                     +1

                                    e1
Genomic   AGGGGGAAGATAGAGATCAAGAAGATAGAGAATCCTACCAACAGGCAGGTGACCTACTCC
cDEF      AGGGGGAAGATAGAGATCAAGAAGATAGAGAATCCTACCAACAGGCAGGTGACCTACTCC 90
tDEF      AGGGGGAAGATAGAGATCAAGAAGATAGAGAATCCTACCAACAGGCAGGTGACCTACTCC 90

                                    e1
Genomic   AAGAGGAGGACGGGGATCATGAAGAAGGCTAAGGAGCTGACGGTGCTTTGCGATGCTGAG
cDEF      AAGAGGAGGACGGGGATCATGAAGAAGGCTAAGGAGCTGACGGTGCTTTGCGATGCTGAG 150
tDEF      AAGAGGAGGACGGGGATCATGAAGAAGGCTAAGGAGCTGACGGTGCTTTGCGATGCTGAG 150

                                    e1
Genomic   GTCTCGCTTATCATGTTCTCCAGCACCGGCAAGTTCTCCGAGTATTGCAGCCCCCTTTCC
cDEF      GTCTCGCTTATCATGTTCTCCAGCACCGGCAAGTTCTCCGAGTATTGCAGCCCCCTTTCC 210
tDEF      GTCTCGCTTATCATGTTCTCCAGCACCGGCAAGTTCTCCGAGTATTGCAGCCCCCTTTCC 210

                                    i1
Genomic   GAGTGTGTACACGATATTATCCCTCCTCGTTCCCCTTTTTTTTTTTTCTTTTTGATAAAA
cDEF      GA---------------------------------------------------------- 212
tDEF      GA---------------------------------------------------------- 212

                                    i1
Genomic   ATGAAACTCATATAGTCTTCTTTTATGATTATGTGTTTGCAATGATGGATGATTGATGGC
cDEF      ------------------------------------------------------------
tDEF      ------------------------------------------------------------

                                                        e2
Genomic   TGGATGGCAGCACCAAGACCATATTTGATCGCTACCAGCAGGTGTCAGGGATCAACCTGT
cDEF      ----------CACCAAGACCATATTTGATCGCTACCAGCAGGTGTCAGGGATCAACCTGT 262
tDEF      ----------CACCAAGACCATATTTGATCGCTACCAGCAGGTGTCAGGGATCAACCTGT 262

                e2                              i2
Genomic   GGAGCGCCCAATACGAGGCAGAAACTCTTCTTCTTCTTCTCCTCTCTCTCTACAAATATG
cDEF      GGAGCGCCCAATACGAG------------------------------------------- 279
tDEF      GGAGCGCCCAATACGAG------------------------------------------- 279

                                    i2
Genomic   CTTTCTTTCTAATTTTTCTTTTCAAAGAAAAAAGAAAAAAAAATGATTTCTAATATTGAT
cDEF      ------------------------------------------------------------
tDEF      ------------------------------------------------------------

                i2                              e3
Genomic   GTATTTTCTTGTGGGAGTAGAAAATGCAAAACACTTTGAACCATCTGAGGGAGATCAACC
cDEF      --------------------AAAATGCAAAACACTTTGAACCATCTGAGGGAGATCAACC 319
tDEF      --------------------AAAATGCAAAACACTTTGAACCATCTGAGGGAGATCAACC 319

                e3                              i3
Genomic   AGAACCTCCGCAGAGAAATAAGGTGGAGGGCCAAAAGAGAATATTGTAATATTAGTACTT
cDEF      AGAACCTCCGCAGAGAAATAAGG------------------------------------- 342
tDEF      AGAACCTCCGCAGAGAAATAAGG------------------------------------- 342

                                    i3
Genomic   TCTGGTAAAAATAAGCATGTAGTTTCTTTTTGCCTTTAAATTTTGTTGTGCTGGTTCTGA
cDEF      ------------------------------------------------------------
tDEF      ------------------------------------------------------------

           i3                       e4
Genomic   TGAGCAGGCAGCGGATGGGTGAAGATCTCGACAGTTTGGGCATCCATGAACTGCGCGGTC
cDEF      --------CAGCGGATGGGTGAAGATCTCGACAGTTTGGGCATCCATGAACTGCGCGGTC 394
tDEF      --------CAGCGGATGGGTGAAGATCTCGACAGTTTGGGCATCCATGAACTGCGCGGTC 394
```

```
                                  e4                            i4
Genomic   TTGAGCAAAATTTAGATGAGGCTTTGAAGGTTGTTCGTCACAGAAAAGTAAGATCCCCCA
cDEF      TTGAGCAAAATTTAGATGAGGCTTTGAAGGTTGTTCGTCACAGAAAA------------- 441
tDEF      TTGAGCAAAATTTAGATGAGGCTTTGAAGGTTGTTCGTCACAGAAAA------------- 441

                                  i4
Genomic   TTTATTCACTGCACCTATTTTAATTCCTTATTCTCCATGTTTTGAGAGCTTTTGAGATAA
cDEF      ------------------------------------------------------------
tDEF      ------------------------------------------------------------

                                  i4
Genomic   ATGATGAGAAGCGCATCGAGATCGAGTTGTCTATATTCTGGAATGATTAATTTTTTAATT
cDEF      ------------------------------------------------------------
tDEF      ------------------------------------------------------------

                                  i4
Genomic   CTCAATTAATGCTGTTTCATTGCTAAATATTCAGCCATATATTTTGTCTCTGCATGGGAT
cDEF      ------------------------------------------------------------
tDEF      ------------------------------------------------------------

                                  i4
Genomic   TTCTATGCTAAAATTCCTCAGATTTCAGCATACAGAATCCATGAGACTTGCCTTGGCTTT
cDEF      ------------------------------------------------------------
tDEF      ------------------------------------------------------------

                                  i4
Genomic   ACCACAAGTACTCCAGAATCAAAATTGTGAAAGAAAAATAGGATAAATCTGGTTAAGCTG
cDEF      ------------------------------------------------------------
tDEF      ------------------------------------------------------------

                                  i4
Genomic   TAATTTATTTACTTACTTTCTATCTATATTAAAATTATTCAGATTATTTTGCAAATTTAT
cDEF      ------------------------------------------------------------
tDEF      ------------------------------------------------------------

                                  i4                            e5
Genomic   GGATATGCTTGAATCACGTATCTGATACTTTCTCTTCATCTGGATGGCAGTACCATGTGA
cDEF      --------------------------------------------------TACCATGTGA 451
tDEF      --------------------------------------------------TACCATGTGA 451

                          e5                              i5
Genomic   TCACCACGCAGACGGATACCTACAAGAAAAAGGCAAGTAAAAGCTAACATGCTTTCTAGC
cDEF      TCACCACGCAGACGGATACCTACAAGAAAAAG---------------------------- 483
tDEF      TCACCACGCAGACGGATACCTACAAGAAAAAGGCAAG-----GCTAACATGCTTTCT--- 503

                                  i5
Genomic   AATATACGATTACCATCATTCTTTACGGTCTTTGATCCGGTTTTGCGTGTCCACTTCTTA
cDEF      ------------------------------------------------------------
tDEF      ----------TACCATCATTCTTTACGGTCTTTGATCCGGTTTTGCGTGTCCACTTCTTA 553
                                          +502

                                  i5
Genomic   CGTAGTCTTTTTCAAACATTCCTATCTAAGACTGAAGGTAATGATTTGCAAAGGAATAGC
cDEF      ------------------------------------------------------------
tDEF      CGTAGTCTTTTTCAAACATTCCTATCTAAGACTGAAGGTAATGATTTGCAAAGGAATAGC 616

                                  i5
Genomic   TTTACTGTTTTCCTCTAAGTAGATGAAATTCTACTCACGTAGAAAGGAGCCATCATAATT
cDEF      ------------------------------------------------------------
tDEF      TTTACTGTTTTCCTCTAAGTAGATGAAATTCTACTCACGTAGAAAGGAGCCATCATAATT 676
                                                         tDEF-p2F

                                  i5
Genomic   GCAGAAAGAATAAAACTGAATGGAATATGAGTAGAATTGTCAAAATCTTGGTTTAAGGGT
cDEF      ------------------------------------------------------------
tDEF      GCAGAAAGAATAAAACTGAATGGAATATGAGTAGAATTGTCAAAATCTTGGTTTAAGGGT 736
```

```
                                         i5
Genomic       TTTAATAGCCAGATGAGAAAGCAACCTACTTTTCTTGAACAACTTGTTTGTGACTGTCTT
cDEF          ------------------------------------------------------------
tDEF          TTTAATAGCCAGATGAGAAAGCAACCTACTTTTCTTGAACAACTTGTTTGTGACTGTCTT 796
                                                                  tDEF-p2R

                                         i5
Genomic       GTTGCTCCCATCTTGCATCTATGATTAGCAAAATATATGATGAATAGATATTCAGATTTG//
cDEF          ------------------------------------------------------------
tDEF          GTTGCTCCCATCTTGCATCTATGAAAAAAAAAAAAAAAAAAAAAAAAAAA----------- 845
              tDEF-p2R

                i5                       e6                           i6
Genomic     //TTGTTGCAGTTGAAGAACTCTAATGAAGCTCACAAAAATTTACTGCATGAACTTGTAAGT
cDEF          ---------TTGAAGAACTCTAATGAAGCTCACAAAAATTTACTGCATGAACTTG----- 529

                                         i6
Genomic       GGAATTAGACGACTCCGTTGTCCTCCATTTTCTTTTATTTTCTTTAAAATCATCTGCCAT
cDEF          ------------------------------------------------------------

                                         i6                           e7
Genomic       TCAAATAGACAGAAAAAAAAGGATTGATTAGCTATTGGGTGCCTCTTGAATTCAGGAAAT
cDEF          --------------------------------------------------------AAAT 533

                                         e7
Genomic       GAAGGACGAGCACCCAGTTTATGGTTTTGTGGATGATGACCCTAGCAACTACGCAGGTGC
cDEF          GAAGGACGAGCACCCAGTTTATGGTTTTGTGGATGATGACCCTAGCAACTACGCAGGTGC 593

                                         e7
Genomic       ACTGGCTCTTGCCAATGGGGCTTCCCACATGTATGCTTTCCGTGTTCAGCCGAGCCAGCC
cDEF          ACTGGCTCTTGCCAATGGGGCTTCCCACATGTATGCTTTCCGTGTTCAGCCGAGCCAGCC 653

                                         e7
Genomic       GAATCTCCATCGAATGGGGTTTGGCTCCCATGACCTGCGCCTTGCTTGATTTTATTGTAG
cDEF          GAATCTCCATCGAATGGGGTTTGGCTCCCATGACCTGCGCCTTGCTTGATTTTATTGTAG 713
                                                            +676

Genomic       CTTAAAGACCTTACAACTTCCAGAGTGGTGTTATATATTAGTATCTTAAGCTATATGACA
cDEF          CTTAAAGACCTTACAACTTCCAGAGTGGTGTTATATATTAGTATCTTAAGCTATATGACA 773

Genomic       GTGGTAAGCCTCTCTATCCGCTACTTGTTATCCTTTAGGTACTTTGCATGTGGTGCAAGG
cDEF          GTGGTAAGCCTCTCTATCCGCTACTTGTTATCCTTTAGGTACTTTGCATGTGGTGCAAGG 833
                      cDEF-p1F

Genomic       TTATAATTGCCTTGTGTTTCTATTGTCTTCCTCATGGTACTTACTGGACTGATGATGTCA
cDEF          TTATAATTGCCTTGTGTTTCTATTGTCTTCCTCATGGTACTTACTGGACTGATGATGTCA 893

Genomic       AGTGAAATGGAGTTGTTTGAATCCTGACTGAAATTTCTCTTGGTCCATCAAGTGCAAGAG
cDEF          AGTGAAATGGAGTTGTTTGAATCCTGACTGAAATTTCTCTTGGTCCATCAAGTGCAAGAG 953
                              cDEF-p1R

Genomic       TAAGTTTAGACATCACTCGCAAGCTTTTGCTAGGAAATAAGTAGTTTCATTGCACTAATG
cDEF          TAAGTTTAGACATCAAAAAAAAAAAAA--------------------------------- 979
```
