# Supplementary material for: DNA Methylation and Expression of the EgDEF1 Gene and Neighboring Retrotransposons in mantled Somaclonal Variants of Oil Palm
Source: PLoS One. 2014 Mar 17;9(3):e91896. doi: 10.1371/journal.pone.0091896 (PMC3956824; doi:10.1371/journal.pone.0091896)
Supplement: Table S1 — Genetic origin of the plant material. Conventionally, crosses are given under the form “male parent x female parent”. (PDF) [file pone.0091896.s009.pdf]

Table S1: Genetic origin of the plant material.

Clonal line	Origin	Pedigree
FC166	Malaysia	D3D x L238T
FC2317	Malaysia	EMB x (L718T x L322P)
FC2318	Malaysia	ELP x (L718T x L718T)
FC2405	Malaysia	ELP1 x BAA5
L2T	Ivory Coast	L2T
LMC51	Ivory Coast	L2T x D8D
LMC343	Ivory Coast	(D115D x D115D) x (L2T x L2T)

Conventionally, crosses are given under the form “male parent x female parent”.
