# Supplementary material for: DNA Methylation and Expression of the EgDEF1 Gene and Neighboring Retrotransposons in mantled Somaclonal Variants of Oil Palm
Source: PLoS One. 2014 Mar 17;9(3):e91896. doi: 10.1371/journal.pone.0091896 (PMC3956824; doi:10.1371/journal.pone.0091896)
Supplement: Table S2 — List of primers used in genome walking and RACE experiments. Primer names beginning with “5” and “3” are primers designed for the amplification of sequences located respectively 5′ or 3′ relatively to their target region. Primer names ending with “n” are used in nested amplifications. (PDF) [file pone.0091896.s010.pdf]

Table S2: List of primers used in genome walking and RACE experiments.

Target region	Primer name	Sequence (5'-3')
downstream from the ATG	3tsDEF-1	GAGACCTTCCTGGGTGCTTGAGAAATAG
	3tsDEF-1n	GGAGATGGGGAGGGGGAAGATAGAGATC
upstream from exon 1	5gDEF-1	CCTGCCTGTTGGTAGGATTCTCTATCTTCTT
	5gDEF-1n	CCCCCTCCCCATCTCCAACTCTCTTTT
downstream from exon 1	3iDEF-1	GATGCTGAGGTCTCGCTTATCATGT
	3iDEF-1n	TCCAGCACCGGCAAGTTCTCCGA
upstream from exon 5	5iDEF-4	CTTTTTCTTGTAGGTATCCGTCTGC
	5iDEF-4n	CCGTCTGCGTGGTGATCACATGGTA
downstream from exon 5	3iDEF-5	TACCATGTGATCACCACGCAGACGG
	3iDEF-5n	GCAGACGGATACCTACAAGAAAAAG
upstream from exon 7	5iDEF-6	CAGTGCACCTGCGTAGTTGCTAGGGTCA
	5iDEF-6n	GGGTCATCATCCACAAAACCATAAACTGGG
downstream from exon 7	3DEFend	CCCAGTTTATGGTTTTGTGGATGATGACCC
	3DEFendn	TGACCCTAGCAACTACGCAGGTGCACTG

Primer names beginning with "5" and "3" are primers designed for the amplification of sequences located respectively 5' or 3' relatively to their target region. Primer names ending with "n" are used in nested amplifications.
