# Supplementary material for: DNA Methylation and Expression of the EgDEF1 Gene and Neighboring Retrotransposons in mantled Somaclonal Variants of Oil Palm
Source: PLoS One. 2014 Mar 17;9(3):e91896. doi: 10.1371/journal.pone.0091896 (PMC3956824; doi:10.1371/journal.pone.0091896)
Supplement: Table S3 — Probes used in Southern blot experiments. The position of each probe relatively to the first coding base of the EgDEF1 gene is indicated in Figure 1. (PDF) [file pone.0091896.s011.pdf]

Table S3: Probes used in Southern blot experiments.

Probe	Size (bp)	Forward primer (5’-3’)	Reverse primer (5’-3’)
P1	786	GTTTTGGGGTCTTCATTAATATACACGGTGG	CCTGCCTGTTGGTAGGATTCTCTATCTTCTT
P2	1900	GATTGGATGTCTCACTATCATCTCGTGAGG	TTGTAGGACTCGAAATGGCTAAGGAGC
P3	702	GGGGTTCAGGGGGTCTTCCAAGAGAGAG	CTGCATGCTCTGTCCCTCAATCATCCGC

The position of each probe relatively to the first coding base of the *EgDEF1* gene is indicated in Figure 1.
