# Supplementary material for: DNA Methylation and Expression of the EgDEF1 Gene and Neighboring Retrotransposons in mantled Somaclonal Variants of Oil Palm
Source: PLoS One. 2014 Mar 17;9(3):e91896. doi: 10.1371/journal.pone.0091896 (PMC3956824; doi:10.1371/journal.pone.0091896)
Supplement: Table S4 — List of primers used in DNA methylation analyses. Primers used to amplify the fragments depicted in Figure 1 are indicated. Primer names beginning with the letter “b” were used in bisulfite sequencing analyses, those beginning with “m” were used in McrBC-PCR analyses. (PDF) [file pone.0091896.s012.pdf]

Table S4: List of primers used in DNA methylation analyses.

Target region	PCR fragment(s)	Primer name	Sequence (5’-3’)
EgDEF1		b1F-DEF	GAGAAGAGAGAGAGTAAGAGAGAAAGGAGG
	F1, F2	b2F-DEF	GGAGGTATAGAGAGTGTGAGATGGGAAAAG
	F3	b3F-DEF	GAGAGTTGGAGATGGGGAGGGGGAAGATAGAG
		b1R-DEF	CTCTATCTTCCCCCTCCCCATCTCCAACTCTC
	F2, F3	b2R-DEF	CTACCACCCAACCATCAATCATCCATCATTAC
		b3R-DEF	ATCTTCACCCATCCACTACCTACTCATCAAAACC
	M1	m1F-DEF	TGGCGAGACATCACACGTTACCCG
	M2	m2F-DEF	GGAAAGTGAAGCCATTATGGAAGCGC
	M3	m3F-DEF	GAGAGTTGGAGATGGGGAGGGGG
	M1	m1R-DEF	CTTGATCTCTATCTTCCCCCTCCCC
	M2	m2R-DEF	GGGGACGAGGAGGGATAATATCGTG
	M3	m4R-DEF	AGGTTGATCCCTGACACCTGCTGG
gypsy retrotransposon (*Koala*)		b1F-gypsy	GATGGAAAAATGTATTGTTGGTATATGTTATATG
	G1	b2F-gypsy	GTTATATGATTTTATTATCTGGATACGTGGTGA
	G2	b3F-gypsy	CCGTAAGAATGCAGACAGTTATTTAATAGCAG
	G3	b4F-gypsy	GTCAGGATTGATAGAGTGGAGATTCTGCTG
	G1	b1R-gypsy	CTGCTATTAAATAACTGTCTGCATTCTTACGG
	G2	b2R-gypsy	ATAAAAAATACCTTACCCCCTTCATATATA
		b3R-gypsy	GCGGATACCTTCTGATTCAAAATCCTC
	G3	b4R-gypsy	ATACCCTTCCTCTAACACTAATCTATTACTG
	M4	m1F-gypsy	GCCACTAACCAACCTCTTAACTAGATGG

	M5	m3F-gypsy	GTTATACTACAGTCAATCACCATGTGGG
	M4	m1R-gypsy	GTTAGACTATTAGGAGAGTTTGTTAGATCG
	M5	m3R-gypsy	CTACCGTTCTATCGTATACTACAGCAG
copia retrotransposon (*Rider*)		b1F-copia	CGAAATAAGGTTGATATTGTTAGAATTTGATG
	C1	b2F-copia	AAAACGGAGTTCGGATGGAGGAG
	C2	b3F-copia	GGGCGGTTCAGGGCTGGGTCTGT
		b4F-copia	GGGTTTGTGGACGAACAGTAAAGTAGAGG
	C3	b5F-copia	GGGGTTCAGGGGGTTTTTAAGAGAGA
	C1	b1R-copia	ACAGACCCAGCCCTGAACCGCCC
	C2	b2R-copia	CCTCTACTTTACTGTTCGTCCACAAACCC
		b3R-copia	CGCCTCCATAAAACATACAAACTTCAC
		b4R-copia	CTCCATCTTCAGTCTTACTCACCACCAC
	C3	b5R-copia	CTGACCAGAACACCACCTCCCAAAATC
	M6	m2F-copia	AATTTTGATGCATAGTCAGGCTGCGAGTC
	M7	m4F-copia	CCTGCCTGGCCCTTTACCCCGGT
	M6	m2R-copia	ACCGGGGTAAAGGGCCAGGCAGG
	M7	m4R-copia	CCTCCATGGTGGTCGGCTTCTCATCACAC

Primers used to amplify the fragments depicted in Figure 1 are indicated. Primer names beginning with the letter “b” were used in bisulfite sequencing analyses, those beginning with “m” were used in McrBC-PCR analyses.
