# Supplementary material for: DNA Methylation and Expression of the EgDEF1 Gene and Neighboring Retrotransposons in mantled Somaclonal Variants of Oil Palm
Source: PLoS One. 2014 Mar 17;9(3):e91896. doi: 10.1371/journal.pone.0091896 (PMC3956824; doi:10.1371/journal.pone.0091896)
Supplement: Table S5 — List of primers used for rt-qPCR. Primer position on their respective target sequence is shown in Figures S6 and S7. (PDF) [file pone.0091896.s013.pdf]

Table S5: List of primers used for rt-qPCR.

Primer position on their respective target sequence is shown in Figures S6 and S7.

Target region	Primer name	Sequence (5'-3')	Product size (bp)
EgDEF1	cDEF-p1F	TGGTAAGCCTCTCTATCCG	152
	cDEF-p1R	TTTCAGTCAGGATTCAAACAACTC	
	tDEF-p2F	CGTAGAAAGGAGCCATCATAAT	156
	tDEF-p2R	AGATGGGAGCAACAAGAC	
gypsy retrotransposon (*Koala*)	RT1-QF5	GAGATCGGGCAAGTCAAGCTA	153
	RT1-QR5	ACTGATGCTTTGGAAGGC	
	RT1-QF1	CTGAAGATGTTCAGGAGAATACCTA	169
	RT1-QR1	GAGGAGTTGGTTGCTAAGTG	
	RT1-QF3	GTTTAGTCAGTCTCTAATATGCCT	156
	RT1-QR3	TTTCAAGAGTTTGTCGACTTCC	
copia retrotransposon (*Rider*)	RT2-QF1	AAGTTCGATGGTAAGAGCAATTT	150
	RT2-QR1	CACCGCCTGCATCTGTA	

RT2-QF3	TATCTACTTCTTTCCAGCAGTGGATATTA	166
RT2-QR3	TGTGTCCTCTAGCTGATCGTC	
