# Supplementary material for: DNA Methylation and Expression of the EgDEF1 Gene and Neighboring Retrotransposons in mantled Somaclonal Variants of Oil Palm
Source: PLoS One. 2014 Mar 17;9(3):e91896. doi: 10.1371/journal.pone.0091896 (PMC3956824; doi:10.1371/journal.pone.0091896)
Supplement: Table S6 — Sequence characteristics of the amplification fragments analyzed through bisulfite sequencing. For the localization of each bisulfite-PCR fragment on the corresponding target region (EgDEF1 gene or retrotransposons), see Figure 1. For the list of primers used to generate these fragments, see Table S5. (PDF) [file pone.0091896.s014.pdf]

Table S6 : Sequence characteristics of the amplification fragments analyzed through bisulfite sequencing.

Target region	PCR fragment	Size (bp)	Number of Cs	CG (%)	CHG (%)	CHH (%)
EgDEF1	F1	338	83	11 (13.3%)	8 (9.6%)	64 (77.1%)
	F2	627	143	20 (14.0%)	19 (13.3%)	104 (72.7%)
	F3	321	60	9 (15.0%)	11 (18.3%)	40 (66.7%)
gypsy retrotransposon (*Koala*)	G1	322	57	9 (15.8%)	18 (31.6%)	30 (52.6%)
	G2	225	51	2 (3.9%)	9 (17.7%)	40 (78.4%)
	G3-a1	327	76	4 (5.3%)	12 (15.8%)	60 (79.0%)
	G3-a2	327	77	6 (7.8%)	12 (15.6%)	59 (76.6%)
copia retrotransposon (*Rider*)	C1	319	105	43 (41.0%)	21 (20.0%)	41 (39.1%)
	C2	209	45	14 (31.1%)	11 (24.4%)	20 (44.4%)
	C3	263	43	5 (11.6%)	8 (18.6%)	30 (69.8%)

For the localization of each bisulfite-PCR fragment on the corresponding target region (*EgDEF1* gene or retrotransposons), see Figure 1. For the list of primers used to generate these fragments, see Table S5.
