# Supplementary material for: DNA Methylation and Expression of the EgDEF1 Gene and Neighboring Retrotransposons in mantled Somaclonal Variants of Oil Palm
Source: PLoS One. 2014 Mar 17;9(3):e91896. doi: 10.1371/journal.pone.0091896 (PMC3956824; doi:10.1371/journal.pone.0091896)
Supplement: Table S7 — Allele-specific analysis of DNA methylation within the G3 region of the Koala retrotransposon. n is the number of individually cloned amplicons analyzed and the average of methylation rates between both alleles is weighted accordingly. (PDF) [file pone.0091896.s015.pdf]

Table S7 : Allele-specific analysis of DNA methylation within the G3 region of the *Koala* retrotransposon.

		% methylated				
Tissue	PCR fragment	CG sites	CHG sites	CHH sites	total methylation (%)	n
N male	G3-a1	100.0	91.6	4.2	23.1	16
	G3-a2	97.9	85.3	3.9	24.1	20
	**average**	**98.8**	**88.1**	**4.1**	**23.7**	**36**
M male	G3-a1	95.0	86.1	4.1	22.0	15
	G3-a2	98.3	81.5	3.3	23.1	16
	**average**	**96.7**	**83.7**	**3.7**	**22.6**	**31**
N female	G3-a1	95.7	90.9	4.8	23.2	16
	G3-a2	99.1	81.1	2.3	22.2	19
	**average**	**97.6**	**85.6**	**3.5**	**22.7**	**35**
M female	G3-a1	97.2	88.9	4.5	22.7	18
	G3-a2	91.7	73.2	1.7	19.74	18
	**average**	**94.5**	**81.1**	**3.1**	**21.22**	**36**

n is the number of individually cloned amplicons analyzed and the average of methylation rates between both alleles is weighted accordingly.
