# Supplementary material for: DNA Methylation and Expression of the EgDEF1 Gene and Neighboring Retrotransposons in mantled Somaclonal Variants of Oil Palm
Source: PLoS One. 2014 Mar 17;9(3):e91896. doi: 10.1371/journal.pone.0091896 (PMC3956824; doi:10.1371/journal.pone.0091896)
Supplement: Table S8 — Sequence characteristics of the amplification fragments analyzed through McrBC-PCR. *McrBC half-sites are two RmC dinucleotides (where R is A or G and mC is a methylated cytosine) separated by 55 to 103 nucleotides in optimal conditions, the cut occurring randomly between them. For the localization of each McrBC-PCR fragment on the corresponding target region (EgDEF1 gene or retrotransposons), see Figure 1. For the list of primers used to generate these fragments, see Table S5. (PDF) [file pone.0091896.s016.pdf]

Table S8: Sequence characteristics of the amplification fragments analyzed through McrBC-PCR.

Target region	PCR fragment	Size (bp)	Number of Cs	Number of potential McrBC half-sites*
EgDEF1	M1	298	77	5
	M2	442	115	5
	M3	369	74	4
gypsy retrotransposon (*Koala*)	M4	509	97	15
	M5-a1	680	151	14
	M5-a2	680	153	13
copia retrotransposon (*Rider*)	M6	749	169	44
	M7	846	173	32

*McrBC half-sites are two RmC dinucleotides (where R is A or G and mC is a methylated cytosine) separated by 55 to 103 nucleotides in optimal conditions, the cut occurring randomly between them. For the localization of each McrBC-PCR fragment on the corresponding target region (*EgDEF1* gene or retrotransposons), see Figure 1. For the list of primers used to generate these fragments, see Table S5.
