# Supplementary material for: DNA Methylation and Expression of the EgDEF1 Gene and Neighboring Retrotransposons in mantled Somaclonal Variants of Oil Palm
Source: PLoS One. 2014 Mar 17;9(3):e91896. doi: 10.1371/journal.pone.0091896 (PMC3956824; doi:10.1371/journal.pone.0091896)
Supplement: Table S9 — Size of intron 5 in orthologs and putative orthologs of the APETALA3 and DEFICIENS genes. The presence of TE-related sequences in the largest intron was assessed using CENSOR (http://www.girinst.org/censor/index.php). (PDF) [file pone.0091896.s017.pdf]

Table S9: Size of intron 5 in orthologs and putative orthologs of the *APETALA3* and *DEFICIENS* genes.

Organism	Gene name	Genomic sequence (size)	mRNA sequence (size)	Largest intron / presence of intronic TE sequences
Oriza sativa japonica	*SUPERWOMAN1* (*SW1*)	AF424549.1 (4.84 kbp)	Gramene LOC_Os06g49890.1 (1,349 nucl)	i5 (2.1 kb) / yes
Zea mays	*SILKY1* (*Si1*)	Maize GDB GRMZM2G139073 (4.68 kbp)	NM_001111481.1 (1,004 nucl)	i5 (2.0 kb) / yes
Sorghum bicolor	*SbMADS16*	NC_012879.1 (4.26 kbp)	XM_002438958.1 (681 nucl)	i5 (2.4 kb) / yes
Brachypodium dystachion	*BdMADS16*	NC_016131.1 (8.07 kbp)	XM_003560499.1 (702 nucl)	i5 (4.7 kb) / yes
Asparagus officinalis	*AoDEF*	AB180962.1 (8.55 kbp)	AB180962.1, annotated CDS (678 nucl)	i5 (4.7 kb) / yes
Arabidopsis thaliana	*APETALA3* (*AP3*)	TAIR10 AT3G54340 (2 kbp)	NM_115294.5 (1,037 nucl)	i5 (275 nucl) / no
Medicago truncatula	*MtAP3-like*	NC_016409.1 (3.85 kbp)	XM_003603673.1 (900 nucl)	i4 (2.5 kb) / yes
Solanum lycopersicum	*TAP3*	NC_015441.1 (3.20 kbp)	NM_001247148.1 (771 nucl)	i4 (803 nucl) / yes
Populus trichocarpa	*PtMADS3*	NW_001492764.1 (1.82 kbp)	XM_002327739.1 (948 nucl)	i4 (332 nucl) / no
Vitis vinifera	*VvAP3*	NC_012024.3 (6.06 kbp)	XM_0022779699.2 (1,211 nucl)	i4 (4.1 kb) / yes

The presence of TE-related sequences in the largest intron was assessed using CENSOR (http://www.girinst.org/censor/index.php).
